# Cortical Responses to Touch Reflect Subcortical Integration of LTMR Signals

**DOI:** 10.1038/s41586-021-04094-x

**Published:** 2021-11-17

**Authors:** Alan J. Emanuel, Brendan P. Lehnert, Stefano Panzeri, Christopher D. Harvey, David D. Ginty

**Affiliations:** 1 Department of Neurobiology, Harvard Medical School, Boston, MA 02115; 2 Howard Hughes Medical Institute, Harvard Medical School, Boston, MA 02115; 3 Department of Excellence for Neural Information Processing, Center for Molecular Neurobiology (ZMNH), University Medical Center Hamburg-Eppendorf (UKE), D-20251 Hamburg, Germany; 4 Neural Computation Laboratory, Istituto Italiano di Tecnologia, 16153 Genova, Italy.

## Abstract

Current models to explain how signals emanating from cutaneous mechanoreceptors generate representations of touch are based on comparisons of the tactile responses of mechanoreceptor subtypes and neurons in somatosensory cortex^1–8^. Here, we used mouse genetic manipulations to investigate the contributions of peripheral mechanoreceptor subtypes to cortical responses to touch. Cortical neurons exhibited remarkably homogeneous and transient responses to skin indentation that resembled rapidly adapting (RA) low-threshold mechanoreceptor (LTMR) responses. Concurrent disruption of signals from both Aβ RA-LTMRs and Aβ slowly adapting (SA-) LTMRs eliminated cortical responses to light indentation forces. However, disruption of either LTMR subtype alone caused opposite shifts in cortical sensitivity but otherwise largely unaltered tactile responses, indicating that both subtypes contribute to normal cortical responses. Selective optogenetic activation of single action potentials in Aβ RA-LTMRs or Aβ SA-LTMRs drove low-latency responses in the majority of mechanically sensitive cortical neurons. Likewise, most somatosensory thalamic neurons were also driven by activation of Aβ RA-LTMRs or Aβ SA-LTMRs. These findings support a model in which signals from physiologically distinct mechanoreceptor subtypes are extensively integrated and transformed within the subcortical somatosensory system to generate cortical representations of touch.

A fundamental question in sensory neuroscience is how signals originating in primary sensory neurons are represented in the cortex and thereby used to generate internal representations of the world. In the somatosensory system, the primary sensory neurons for light touch of glabrous (non-hairy) skin include Aβ RA-LTMRs that innervate either Meissner or Pacinian corpuscles and Aβ SA-LTMRs that either form associations with Merkel cells or, in some species, may form Ruffini endings^9,10^. The contributions of these mechanoreceptor subtypes to cortical representations have been inferred by correlative comparisons of LTMR and cortical responses to mechanical stimuli^1–8^, but functional perturbation experiments that test how the signals from individual Aβ LTMR subtypes generate cortical representations have not been performed. We therefore used selective genetic and optogenetic manipulations to eliminate or activate Aβ LTMR subtypes while recording responses in S1 and somatosensory thalamus.

Because tactile responses of neurons within forepaw and hindpaw regions of mouse S1 have not been studied in depth, we began by comparing tactile response properties of neurons in S1 to those of primary cutaneous Aβ LTMRs. We recorded directly from cutaneous Aβ LTMRs in an anesthetized, *in vivo* preparation^11^ while stimulating glabrous skin with step indentations of intensities that span the expected thresholds of both low- and high-threshold mechanoreceptors^12–15^. Aβ LTMRs with RA responses (action potentials produced only during the onset and/or offset of indentations) and SA responses (action potentials at the onset and throughout the indentation period) were present in approximately equal numbers^9,12–14^ (Fig. 1a and Extended Data Fig. 1a). Aβ RA-LTMRs and Aβ SA-LTMRs exhibited localized receptive fields (Extended Data Figs. 1b,c) and had comparable sensitivities (Extended Data Fig. 1d). Furthermore, transient and sustained phases of their responses approached saturation at indentation forces between 20 and 40 mN (Fig. 1a). We also assessed tactile responses of Aβ LTMRs that innervate Pacinian corpuscles in ankle and digit joints. While 100-Hz vibration activated Pacinian Aβ LTMRs when applied to glabrous skin of the paw (Extended Data Fig. 1e–g), step indentations did not (Extended Data Fig. 1f). In total, the force steps we applied to glabrous skin activate comparable numbers of Aβ RA-LTMRs of Meissner corpuscles and Merkel cell-associated Aβ SA-LTMRs but do not activate Pacinian corpuscle-associated Aβ LTMRs.

To assess cortical responses, we used multielectrode array electrophysiology in S1 of paw-tethered, awake mice (Extended Data Fig. 2) and focused on passive response properties by excluding trials during which the mouse moved its stimulated paw (Extended Data Fig. 3, Methods). We applied 10-mN step indentations in a grid to measure receptive fields (RFs) of hindpaw and forepaw S1 units (Extended Data Figs. 4a,g). Consistent with measurements in rats^16,17^, stimuli at many locations across the ventral paw increased the firing rates of individual units in mouse S1 (Extended Data Fig. 4), even for excitatory layer IV neurons identified by optotagging (Extended Data Figs. 2b–e,4f). Because RFs were often noncontiguous, we used information theoretic analysis to evaluate RFs agnostic to their structure (Extended Data Fig. 4). Mutual information between the stimulus location and neural response (spatial information) was apparent at the indentation step onset and step offset but not during the sustained phase (Extended Data Figs. 4b,c,h,i). Hindpaw and forepaw S1 RFs were similar in size, and S1 RFs were larger than those of glabrous hindpaw-innervating Aβ SA- and RA-LTMRs. This indicates that mechanical properties of the skin cannot account for the expanse of cortical RFs and instead that signals from multiple peripheral mechanoreceptors converge upon individual cortical neurons.

We next assessed response profiles and intensity-response relationships using a series of step indentations from 1 to 75 mN. Typical hindpaw S1 units responded to step indentations of glabrous skin at the onset and offset of the step but rarely to the sustained portion of the step (Fig. 1b,c and Extended Data Fig. 5d). In fact, while the firing rates at the onset and offset of steps were markedly higher than baseline firing rates at intensities as low as 5 mN, the firing rates during the sustained portion of the step were indistinguishable from the baseline firing rates, except at the highest intensity (Fig. 1d). This transient response profile was similar across cortical layers and in both fast-spiking (FS) and regular-spiking (RS) units (Figs. 1b,c and Extended Data Fig. 5d). The few hindpaw S1 units with sustained responses to 75-mN step indentations were distributed throughout the cortical depth and across RS and FS units. Therefore, the response profiles of hindpaw S1 units are predominantly transient and homogeneous across cortical cell types and laminar location.

Forepaw S1 was also largely comprised of units with transient responses at step onsets and offsets (Extended Data Figs. 6a–e). However, a larger fraction of forepaw S1 units exhibited sustained increases in firing during high force indentations. Notably, these forepaw S1 sustained responses emerge at or above the force required to saturate the sustained response of Aβ SA-LTMRs, which suggests that sustained responses from Aβ SA-LTMRs do not contribute to S1 responses or are selectively filtered at low intensities to produce transient S1 responses.

In both hindpaw and forepaw S1, transient responses at the onset and offset of the step indentation grew with stronger forces until they saturated, typically around 40 mN, similar to saturation of all response phases observed in Aβ LTMRs (Fig. 1a). The intensity-response relationships for many S1 units were correspondingly fit well with a saturating exponential (examples in Extended Data Figs. 5e,f). For hindpaw and forepaw S1, there were no differences in the fit parameter *I*_*0*_ (a measure of sensitivity) across layers or between well-fit RS and FS units, but forepaw S1 units were more sensitive than hindpaw S1 units (Extended Data Fig. 5g).

Overall, while comparable numbers of Aβ RA-LTMRs and Aβ SA-LTMRs with similar sensitivity and small receptive fields are activated by step indentations of the hindpaw, the corresponding responses of hindpaw and forepaw S1 units are strikingly homogeneous. To estimate potential contributions from each LTMR subtype, we fit S1 response profiles as a linear mixture of signals from Aβ RA-LTMRs and Aβ SA-LTMRs, similar to a model used for macaque S1^8^. Nearly all S1 units had weights attributed almost exclusively to the Aβ RA-LTMR profile (Extended Data Figs. 6g,h). Therefore, S1 responses closely resemble Aβ RA-LTMR responses in that the vast majority of units respond transiently to step indentations, with increased firing at the onset and offset of the indentation but not during the sustained phase.

To assess the necessity of Aβ RA-LTMR and Aβ SA-LTMR signals for S1 responses, we employed genetic ablation strategies in separate mice that resulted in: 1) the loss of Meissner corpuscles and their associated pairs of Aβ LTMR endings^14^ (*Advillin*^*Cre*^*;TrkB*^*flox/flox*^, hereafter referred to as *TrkB*^*cKO*^; Figs. 2a–d); 2) the loss of Merkel cells that are required for normal Aβ SA-LTMR responses^18^ (*K5-Cre;Atoh1*^*flox/flox*^, hereafter referred to as *Atoh1*^*cKO*^; Figs. 2e–h); and 3) double knockouts (*Advillin*^*Cre*^*;TrkB*^*flox/flox*^*;Atoh1*^*flox/flox*^, hereafter referred to as DKO) that lack both Meissner corpuscles and Merkel cells (Figs. 2i–l; Extended Data Figs. 7a–d).

Multiple measures of sensitivity, either of the population (fractions of units responding at each intensity) or of individual units (fitted I_0_ values), indicated that hindpaw S1 units were less sensitive in DKOs than in littermate controls and wild-type animals. In fact, no responses were apparent to indentation forces less than 20 mN in DKOs, indicating that Meissner corpuscle- and Merkel cell-associated LTMRs are required for S1 responses to light forces. However, the fraction of DKO S1 units responding to high forces is similar to that in control and wild-type mice (Fig. 2k).

S1 sensitivity was also diminished, but to a lesser degree, in *TrkB*^*cKO*^ mice that only lack Meissner corpuscles. The responses of S1 units from *TrkB*^*cKO*^ mice, especially the transient portion of the response at the offset of the step indentation, were less sensitive than S1 units in *TrkB*^*fl/fl*^ controls and wild-type animals (Figs. 2c,d). Remarkably, some units continued to respond to the indentation offset, even at 10 mN. Because OFF responses were absent in DKO mice at 10 mN (Figs. 2i–k), signals from Aβ SA-LTMRs must be transformed in *TrkB*^*cKO*^ mice to produce the OFF response. In contrast to the *TrkB*^*cKO*^, both the population and individual S1 units of *Atoh1*^*cKO*^ mice, which only lack Merkel cells, exhibited increased sensitivity compared to littermate controls or wild-type animals at both the onset and offset of the step indentations (Figs. 2e–h).

Strikingly, the transient nature of S1 responses to low-intensity steps was largely unaltered in knockout mice (Figs. 2b,f,j). Transient responses were present even in the *TrkB*^*cKO*^ at 10 mN, when the only contribution to cortical responses is from Aβ SA-LTMRs. There were only small differences in response durations at the step onset and offset between S1 units in knockout and control mice (Extended Data Figs. 7e,f). Furthermore, at the highest forces, a slightly larger fraction of S1 units in single knockout mice produced sustained responses than wild-type mice (Figs. 2c,g).

Similarly, RF spatial information at the onset of the 10-mN step was unaltered between single knockouts and their littermate controls (Extended Data Figs. 7g–j). However, spatial information was absent at the offset of the step response for *TrkB*^*cKO*^ S1 units (Extended Data Fig. 7h). There was a slight but significant decrease in the RF area in *TrkB*^*cKO*^ mice compared to littermate controls and wild-type mice (Extended Data Fig. 7h), but the spatial information and the RF size did not differ between *Atoh1*^*cKO*^ mice lacking Merkel cells, *Atoh1*^*fl/fl*^ littermate controls, and wild-type animals (Extended Data Fig. 7i). We assessed RFs at 40 mN in DKOs due to their diminished sensitivity. The RF was smaller, and the mean spatial information was slightly reduced at the step onset and more dramatically reduced at the step offset in DKOs compared to littermate controls (Extended Data Fig. 7j). Together, these findings show that input from both Aβ RA- and SA-LTMRs contribute to the normal response to step indentations for the vast majority and perhaps all S1 units, supporting the idea that the signals from these LTMR subtypes are integrated within S1 or subcortically.

The developmental ablation experiments suggest that both Aβ RA-LTMRs and Aβ SA-LTMRs contribute to normal responses of most if not all S1 neurons. To complement the loss-of-function experiments, we used optogenetic manipulations to test the sufficiency of signals emanating from Aβ LTMR subtypes to modulate the firing rate of S1 units. In separate animals, we expressed ReaChR in either of the two Aβ LTMRs that innervate the Meissner corpuscle or in the Aβ SA-LTMRs that innervate Merkel cells by using a recombinase-dependent ReaChR mouse line^19^ (*R26*^*LSL-FSF-ReaChR*^ or *R26*^*LSL-ReaChR*^). Recombination of the Ret^+^ and TrkB^+^ Meissner corpuscle Aβ LTMRs was selectively driven by tamoxifen-inducible *Ret*^*CreER*^ (these mice hereafter referred to as *Ret::ReaChR*) and *TrkB*^*CreER*^ (hereafter referred to as *TrkB::ReaChR*) recombinase driver lines^14,20^, respectively, whereas ReaChR expression in Aβ SA-LTMRs was achieved using the *TrkC*^*CreER*^ (hereafter referred to as *TrkC::ReaChR*) recombinase driver line^11^. For all three lines, only axons of the large-diameter sensory neurons of interest were labeled within glabrous skin of the paws (Extended Data Fig. 8a).

We optically activated Aβ LTMR subtypes by focally flashing light onto the skin at randomized locations within an 8 mm square centered on the pedal pads. In ReaChR-expressing Aβ LTMRs, pulses of light directed onto the mechanical RF reliably generated a short-latency single action potential (Fig. 3a and Extended Data Figs. 8b–d). ReaChR-expressing proprioceptors (driven by *TrkC*^*CreER*^, the driver line we used for Aβ SA-LTMRs) were not activated (Extended Data Fig. 8e). Therefore, this stimulation paradigm selectively evokes single action potentials in cutaneous, ReaChR-expressing Merkel cell-associated Aβ SA-LTMRs or Meissner corpuscle-associated Aβ LTMRs.

We targeted multielectrode arrays to forepaw or hindpaw S1 and measured responses to the same optical stimuli. Strikingly, the majority of S1 units that responded to step indentations of glabrous skin also exhibited increased firing rates within 25 ms of selective optical stimulation of either of the two Meissner corpuscle-associated Aβ LTMRs or Merkel cell-associated Aβ SA-LTMRs (Figs. 3b–d). Therefore, activation of single action potentials in either Aβ RA-LTMRs or Aβ SA-LTMRs is sufficient to drive cortical responses. Latencies were short in superficial layers and longer in deeper layers (17.5 ± 6.7 ms and 22.4 ± 8.7 ms for layer IV and layer V S1 units, respectively [mean ± s.d.]), and the latencies in layer IV were only ~12 ms longer than those measured in the DRG (Fig. 3e). In control experiments, S1 units in mice with ReaChR expression restricted to proprioceptors or in *R26*^*LSL-FSF-ReaChR*^ mice without Cre recombinase expression did not respond to optical skin stimulation (Extended Data Figs. 8f–h). Grand means of the mechanical responses were similar between units driven by optical activation of Meissner corpuscle-associated Aβ LTMRs, units driven by activation of Aβ SA-LTMRs, and units in wild-type mice (Extended Data Fig. 9a). Thus, Ab RA-LTMRs and Aβ SA-LTMRs did not appear to drive S1 neurons with specialized response profiles.

The optical stimulation paradigm enabled a quantitative assessment of the impact of action potentials in the periphery on changes in spiking in S1. In the DRG, activation of Aβ LTMR subtypes near the RF center resulted in 0.55 to 0.97 action potentials per trial. Because the skin is homotypically tiled for both Meissner-corpuscle innervating neurons and Merkel-cell-innervating neurons^14,21^, each light pulse should only alter the spiking of a few Aβ LTMRs. For S1 units, we calculated the number of evoked action potentials in each trial where the laser pulse occurred near the most responsive region. On average, this number in individual S1 units was smaller than in the least responsive Aβ LTMR (Fig. 3f). Activation of the two Aβ LTMRs that innervate Meissner corpuscles in forepaw S1 units resulted in similar numbers of action potentials in S1, whereas activation of Aβ SA-LTMRs that innervate Merkel cells resulted in fewer (Fig. 3f).

While the optical stimulus evoked fewer action potentials in individual S1 neurons than DRG neurons, LTMR signals affect many neurons as they ascend the somatosensory pathway. We estimate that approximately 4,000 neurons in layers II/III to V of forepaw S1 have overlapping mechanical receptive fields (Methods). Thus, we predict, on average, a single action potential in a few TrkB^+^ Aβ RA-LTMRs, Ret^+^ Aβ LTMRs, or TrkC^+^ Aβ SA-LTMRs leads to approximately 1250, 1900, or 450 action potentials, respectively, across the population of S1 neurons. Overall, the signals from the three Aβ LTMR subtypes converge onto most S1 neurons and lead to large amplification of evoked spikes in cortex relative to the DRG, but the extent of this amplification differs across subtypes.

We next asked whether the integration of Aβ RA-LTMR and Aβ SA-LTMR signals occurs within S1 or is inherited from subcortical areas by delivering mechanical and optogenetic stimuli while measuring response properties from the somatosensory thalamus (VPL). We targeted a multielectrode array to the middle of VPL, and the probe position was verified physiologically by monitoring responses to brushing across skin regions and anatomically by post-hoc histology (Extended Data Fig. 9b).

The RFs of forepaw glabrous skin VPL units were similar to those of S1 units, both in spatial information and RF area (Extended Data Figs. 9c–g). The sensitivity of VPL units was on average lower than that of S1 units, but the full cortical sensitivity range is encoded within VPL (Extended Data Figs. 9i,j). Furthermore, the sensitivity and response profiles of VPL units were considerably more heterogeneous than their S1 counterparts (Fig. 4a). While many VPL units exhibited transient responses that resembled those in S1 (Extended Data Fig. 10a), others produced responses that we rarely, if ever, observed within S1, including robust sustained responses (Extended Data Fig. 10b) and decreases in firing rate in response to mechanical stimulation (Extended Data Fig. 10c). The response differences between thalamus and cortex suggest the thalamocortical synapse or circuitry intrinsic to cortex transforms temporally diverse thalamic response profiles into homogeneous and transient S1 responses, perhaps through feedforward inhibition recruited at the thalamocortical synapse^22,23^.

As for S1 recordings, we selectively activated either Aβ RA-LTMRs (using *TrkB::ReaChR* mice) or Aβ SA-LTMRs (*TrkC::ReaChR* mice) with optical stimuli applied to forepaw glabrous skin. If signals from LTMR subtypes converge prior to S1, the majority of VPL neurons would be modulated with optical activation at latencies shorter than those in S1. Indeed, optical stimulation of Aβ RA-LTMRs or Aβ SA-LTMRs drove responses, respectively, in 69% and 72% of the units responsive to 20-mN indentations (Fig. 4b,c). These proportions are lower bound estimates due to incomplete labeling efficiency of the inducible Cre recombinase driver lines. These optical responses in VPL units exhibited shorter latencies than in S1 units (Fig. 4d and Extended Data Fig. 10d), indicating that convergence occurs within the feedforward pathway. Unlike S1, the number of evoked action potentials per light pulse did not differ between activation of Aβ RA-LTMRs and Aβ SA-LTMRs (Fig. 4e), suggesting the difference between subtypes arises at the thalamocortical synapse or within S1. Strikingly, optically evoked responses were observed across the variety of response profiles in VPL (Fig. 4b and examples in Extended Data Figs. 10a–c). We clustered VPL units based on response profiles, and the firing rates of units in each cluster were modulated by selective optogenetic activation of either Aβ RA-LTMRs or Aβ SA-LTMRs (Extended Data Figs. 10e,f). Therefore, both Aβ RA-LTMRs and Aβ SA-LTMRs exert broad influence over VPL neurons, and the signals from distinct Aβ LTMR subtypes converge subcortically.

Overall, our results reveal that, despite a near homogeneous response to step indentations that most closely resembles the responses of Aβ RA-LTMRs, the cortical representation of light touch reflects extensive subcortical integration of signals originating from both Aβ RA-LTMRs and Aβ SA-LTMRs. Previous studies inferred the contributions of mechanoreceptor subtypes to cortical representations by comparing responses of Aβ LTMRs and S1 neurons^1–8^. These studies in some cases concluded that the signals from each LTMR subtype remain segregated in ascending somatosensory pathways and contribute to a select subpopulation of cortical neurons^2,5,6^. In another case, it has been observed that a subset of individual neurons in macaque S1 have responses that resemble both Aβ RA-LTMRs (a transient response at both the onset and offset of indentation) and Aβ SA-LTMRs (a sustained response), leading to the proposal that signals from Aβ LTMR subtypes are linearly combined in a subset of cortical neurons while being maintained separately in other S1 neurons^8^. Both sets of studies imply that Aβ LTMR signals propagate through the somatosensory hierarchy without filtering or transformation.

Our causal manipulations in mice best support a model of somatosensory information processing in which S1 responses to tactile stimuli reflect extensive subcortical convergence and nonlinear transformation of signals emanating from distinct Aβ LTMR subtypes. First, virtually all S1 response profiles were similar in single knockouts that disrupted signaling from either Aβ RA-LTMRs or Aβ SA-LTMRs, and selective optogenetic activation of Aβ RA-LTMRs or Aβ SA-LTMRs drove the majority of S1 and VPL units. Second, selective activation of Aβ SA-LTMRs was sufficient to modulate spiking of S1 (and VPL) units that responded transiently to step indentations. Therefore, the sustained signals generated by Aβ SA-LTMRs must be truncated or otherwise filtered as they ascend the somatosensory pathway. Third, S1 units in mutants lacking both Meissner corpuscles and Merkel cells did not respond to 10-mN indentations, yet S1 units in mice lacking Meissner corpuscles but not Merkel cells exhibited an OFF response to 10-mN step indentations, suggesting that Aβ SA-LTMR responses can also be transformed to generate responses at the step offset. Fourth, the single knockouts shifted S1 sensitivity in opposite directions, indicating that the signals generated by Aβ RA-LTMRs and Aβ SA-LTMRs differentially recruit subcortical circuit elements that set S1 sensitivity.

There are multiple sites at which signals originating from Aβ LTMRs and other DRG neuron types may be transformed and integrated prior to reaching cortex. Indeed, interneurons in the spinal cord are important for normal tactile behavior, and inputs into the spinal cord from distinct LTMR subtypes overlap anatomically^24,25^, suggesting that signal integration occurs as early as the first synapse in the somatosensory pathway. We propose that the extensive subcortical convergence of signals from peripheral mechanoreceptors provides the elements needed for the central encoding of complex features of the physical world including object shape and orientation, texture, movement speed and direction, vibration, and compliance^26–30^.

## Methods

### Animals

All experimental procedures were approved by the Harvard Medical School Institutional Care and Use Committee and were performed in compliance with the Guide for Animal Care and Use of Laboratory Animals. Mice were housed in temperature- and humidity-controlled facility in a 12h:12h light:dark cycle and recordings were performed during the light cycle. S1 and VPL recordings were made from mice between four and twelve weeks of age and included mice with the following genotypes (number of animals in parentheses): C57Bl/6J (9), *Scnn1a-tg3-Cre;R26*^*LSL-ChR2-EYFP/+*^ (5), *TrkB*^*fl/fl*^ (3), *Advillin*^*Cre*^*;TrkB*^*fl/fl*^ (5), *Atoh1*^*fl/fl*^ (3), *K5*^*Cre*^*;Atoh1*^*fl/fl*^ (3), *TrkB*^*fl/+*^*;Atoh1*^*fl/+*^ (1), *TrkB*^*fl/+*^*;Atoh1*^*fl/fl*^ (1), *Advillin*^*Cre*^*;TrkB*^*fl/+*^*;Atoh1*^*fl/+*^ (1)*, Advillin*^*Cre*^*;TrkB*^*fl/fl*^*;Atoh1*^*fl/fl*^ (4), *Ret*^*CreERT2*^*;Advillin*^*FlpO*^*;R26*^*LSL-FSF-ReaChR*^ (8), *TrkC*^*CreERT2*^*;Advillin*^*FlpO*^*;R26*^*LSL-FSF-ReaChR*^ (10), *TrkC*^*CreERT2*^*;R26*^*LSL-ReaChR*^ (2), *TrkB*^*CreERT2*^*;Advillin*^*FlpO*^*;R26*^*LSL-FSF-ReaChR*^ (3)*, TrkB*^*CreERT2*^*;R26*^*LSL-ReaChR*^ (4), *Advillin*^*FlpO*^*;R26*^*LSL-FSF-ReaChR*^ (1), and *Cux2*^*CreERT2*^*;PV*^*2a-FlpO*^*;R26*^*LSL-FSF-ReaChR*^ (3). All alleles have been described previously^11,19,20,31–37^. All mice other than wild-type mice were maintained on mixed C57Bl/6J and CD1 backgrounds and included both male and females. Wild-type C57Bl/6J mice were all males and obtained from Jackson Laboratories (000664). *Cux2*^*CreERT2*^, RRID:MMRRC_032779-MU, was obtained from the Mutant Mouse Resource and Research Center (MMRRC) at University of Missouri and was donated to the MMRRC by Ulrich Mueller, Ph.D., The Scripps Research Institute. DRG recordings were performed on a subset of these mice.

To achieve specific labeling of Aβ LTMR subtypes or proprioceptors^11,14^, CreER driver lines were induced by administering tamoxifen dissolved in sunflower seed oil embryonically via oral gavage to the dam or early postnatally by IP injection. For *Ret*^*CreERT2*^*;Advillin*^*FlpO*^*;R26*^*LSL-FSF-ReaChR*^, we administered 3 mg at embryonic day 11.5 (E11.5), for *TrkC*^*CreERT2*^*;Advillin*^*FlpO*^*;R26*^*LSL-FSF-ReaChR*^ and *TrkC*^*CreERT2*^*;R26*^*LSL-ReaChR*^, 3 mg at E12.5, for *TrkB*^*CreERT2*^*;R26*^*LSL-ReaChR*^, 3 mg at E13.5, for *TrkB*^*CreERT2*^*;Advillin*^*FlpO*^*;R26*^*LSL-FSF-ReaChR*^, 0.5 mg at postnatal day 3 (P3), and for *Cux2*^*CreERT2*^*;PV*^*2a-FlpO*^*;R26*^*LSL-FSF-ReaChR*^, 0.5 mg at P6.

Most DKOs (*Advillin*^*Cre*^*;TrkB*^*flox/flox*^*;Atoh1*^*flox/flox*^) were behaviorally indistinguishable from littermate controls in the home cage. However, a minority of DKOs (one of four included in this study) and some littermates with *Advillin*^*Cre*^*;TrkB*^*flox/+*^*;Atoh1*^*flox/flox*^ genotypes exhibited an uncoordinated gait and cerebellar hypoplasia, consistent with sporadic Cre-mediated recombination at the *Atoh1*^*flox*^ allele in the rhombic lip^38^. There were no systematic differences between S1 responses in the uncoordinated DKO and those in the coordinated DKOs, suggesting the feedforward somatosensory system remained intact in all DKOs.

### Craniotomy

Prior to surgery, mice were treated with dexamethasone (2 mg/kg IP) to prevent swelling and slow-release buprenorphine (0.5–1.0 mg/kg SQ) for analgesia. Mice were anesthetized with 1.5–2% isoflurane. The scalp was removed, the skull was dried, and a titanium headplate was affixed to the skull using dental cement (Metabond). An oval craniotomy (approximately 1.5 mm major axis and 1 mm minor axis) was made that spanned hindpaw and forepaw S1 (targeting coordinates were 0.60 mm posterior and 1.65 mm lateral to bregma and 0.00 mm posterior and 2.10 mm lateral to bregma for hindpaw S1 and forepaw S1, respectively). The same cortical coordinates for hindpaw S1 were used across conditional knockout mice, littermate controls, and wild-type C57Bl/6J mice. The conserved location of hindpaw S1 across these animal models suggests that the overall structure of S1 is preserved despite the loss of signals originating from select LTMR subtypes.

Once the brain was exposed, it was submerged in a HEPES-buffered saline solution (pH 7.4) consisting of (in mM) 150 NaCl, 2.5 KCl, and 10 HEPES. Once hemostasis was achieved, the craniotomy was sealed with Kwik Sil (WPI) and an aluminum ring was cemented onto the headplate to provide a well for a recording bath solution. Mice recovered for at least 24 h prior to recording sessions.

### *In Vivo* Multielectrode Array Electrophysiology

Prior to each recording, the animal was habituated to the recording environment and head fixation for 10–15 min. Then, Kwik Sil covering the craniotomy was removed and the craniotomy was submerged in HEPES-buffered saline solution. A 32-channel silicon probe (Neuronexus A1×32-Poly2-5mm-50s-177-A32 or A1×32-Poly2-5mm-50s-177-OA32 for optotagging) coated with DiI (D3911, Thermo Fisher) was inserted into hindlimb or forelimb S1 and the tip of the probe was advanced to 1100 μm below the dura for S1 recordings or ~4000 μm below the dura for VPL recordings. The saline solution was replaced with 1% agarose (dissolved in HEPES-buffered saline solution) to stabilize the probe and provide a bath for the ground electrode. Additional saline was applied every 30 min to keep the agarose moist. Recordings were amplified, filtered (0.1 – 7.5 kHz bandpass), and digitized (20 kHz) using a headstage amplifier and recording controller (Intan Technologies RHD2132 and Recording Controller). Data acquisition was controlled with open-source software (Intan Technologies Recording Controller version 2.07).

**Extended Data Fig. 1:**
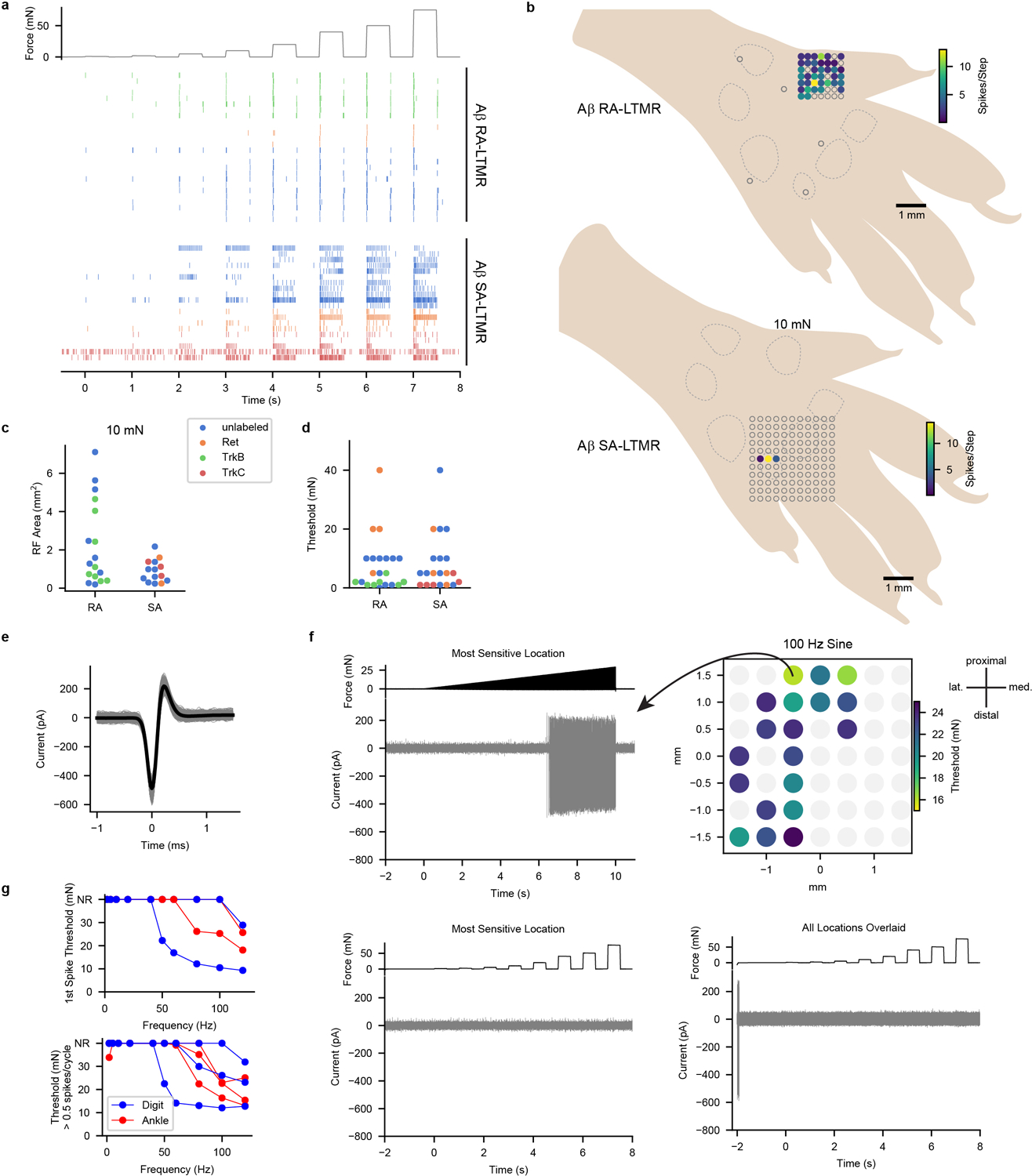
Aβ LTMR Responses to Force-Controlled Step Indentations **a**, Raster plot showing cutaneous Aβ RA-LTMR and Aβ SA-LTMR responses to a series of step indentations ranging from 1 to 75 mN applied to the most responsive skin region for each neuron. Markers are colored according to how the neurons were labeled (Blue, unlabeled; Orange, Ret^+^; Green, TrkB^+^; Red, TrkC^+^). A subset of these recordings (unlabeled neurons that were recorded in littermate controls for *TrkB*^*cKO*^ mice, Ret^+^ neurons, and TrkB^+^ neurons) were previously published^14^. **b**, Example RFs of an Aβ RA-LTMR (top) and an Aβ SA-LTMR (bottom) to 10-mN step indentations superimposed on a schematic of the hindpaw. Dashed lines outline pedal pads. Unfilled markers represent stimulus locations that did not evoke a response. Color represents the total number of action potentials evoked during the step indentation. **c**, RF sizes for Aβ RA-LTMRs (n = 17) and Aβ SA-LTMRs (n = 14) that were responsive to 10-mN step indentations. Markers are colored according to how the neurons were labeled. Mean ± s.e.m. areas of 2.3 ± 0.5 and 0.9 ± 0.2 mm^2^ for Aβ RA-LTMRs and Aβ SA-LTMRs, respectively and median ± i.q.r. of 1.3 ± 3.4 and 0.8 ± 0.9 for Aβ RA-LTMRs and Aβ SA-LTMRs, respectively (Two-sided Mann-Whitney U= 81.0, p=0.07). **d**, Force threshold for step indentation response for Aβ RA-LTMRs (n = 25) and Aβ SA-LTMRs (n = 20). Markers are colored according to how the neurons were labeled. Mean ± s.e.m. thresholds of 9.0 ± 2.1 and 9.2 ± 2.1 mN for Aβ RA-LTMRs and Aβ SA-LTMRs, respectively and median ± i.q.r. of 5.0 ± 8.0 and 5.0 ± 11.5 for Aβ RA-LTMRs and Aβ SA-LTMRs, respectively (Two-sided Mann-Whitney U=253.5, p=0.45). **e**, Individual (gray) and mean (black) waveforms recorded from a Pacinian corpuscle-innervating Aβ LTMR labeled with a *Ret*^*CreER*^;*PV*^*FlpO*^ intersectional strategy (3 mg tamoxifen administered at embryonic day 11.5). These neurons were not labeled with dye-conjugated CTB, which was injected into the pedal and digit pads 48 h prior to recording. **f**, A 100-Hz sine ramp stimulus was applied to multiple locations across the glabrous hindpaw to assess the responsive region for the Pacinian corpuscle-innervating Aβ LTMR. Top left: Response of neuron to most sensitive region. Top right: sine stimulus response threshold for each probed location. This Aβ LTMR likely innervated a Pacinian corpuscle in the ankle joint. Bottom left: Response to step indentations at most sensitive location. Bottom right: Response to step indentations at all locations overlaid. In some locations action potentials are generated as the probe initially comes into contact with the skin but are never generated in response to the step indentations (which were low-pass filtered at 33 Hz). **g**, Frequency-response relationships for sine stimuli delivered in a ramp (top) or in a 1-s step (bottom). All Pacinian corpuscle-innervating Aβ LTMRs were most sensitive to high frequency stimulation. Ankle and digit terminal locations were inferred based on the regions of the paw that responded to a handheld vibrating metal probe. Ankle neurons (n = 3) responded when the probe was applied to most regions of the paw, including digits and pedal pads. Digit neurons (n = 3) only responded when the probe was applied to a single digit.

Shortly after the probe was inserted into the brain, we searched for receptive fields (RFs) by gently brushing the skin of the mouse with a fine paintbrush while listening to spikes from multiple channels. This manual probing revealed the rough location of the RF. If the RF was not on the glabrous paw, the probe was removed from the brain, moved to a new location within the craniotomy, and reinserted. Otherwise, the paw was tethered over a circular aperture (7.6 mm and 6.4 mm diameters for hindpaw and forepaw, respectively) in an acrylic platform that supported the mouse. A 0.5-mm diameter, cylindrical, Teflon-tipped indenting probe was controlled by a dual-mode force controller (Aurora Scientific 300C-I) and was used to stimulate the paw through the aperture. For assessing RF structure, the position of the indenter was controlled with two linear translation piezo stages and a stage controller (Physik Instrumente U-521.24 and C-867.2U2). The position, force, and displacement of the indenter were commanded with custom Matlab (version 2017a) scripts controlling a Nidaq board (National Instruments, NI USB 6259). Force steps were applied atop the minimal force required to keep the indenting probe in contact with the skin. Most Aβ LTMRs did not respond to this holding force (Extended Data Fig. 1a) and no mutual information was present between the stimulus location and cortical activity during baseline periods in which this minimal force was applied to the skin.

### Spike Sorting

We used open-source software^39^ (JRCLUST version 3.2.2) to automatically sort action potentials into clusters, manually refine those clusters, and classify them as single or multi units. The voltage traces were filtered with a differentiation filter of order 3. Frequency outliers were removed with a threshold of 10 median absolute deviations (MADs). Action potentials were detected with a threshold of 4.5 times the standard deviation of the noise. Action potentials with similar times across sites within 60 μm were merged and action potentials were then sorted into clusters with a density-based-clustering algorithm^40^ (clustering by fast search and find of density peaks) with cutoffs for log_10_(ρ) at −3 and log_10_(δ) at 0.6. Clusters with a waveform correlation greater than 0.99 were automatically merged. Outlier spikes (> 6.5 MADs) were removed from each cluster.

The clusters were manually curated with JRCLUST split and merge tools and classified as single or multi units. To qualify as a single unit, the following criteria had to be met: 1) > 99.5% of action potentials were required to have interspike intervals > 2 ms, 2) > 95% of action potentials in the cluster had to be estimated to be greater than the detection threshold based on the mean and standard deviation of their amplitudes, and 3) the waveform had to be distinct from other nearby clusters. Only clusters classified as single units were included in this study.

### Laminar and Cell Type Identification

**Extended Data Fig. 2:**
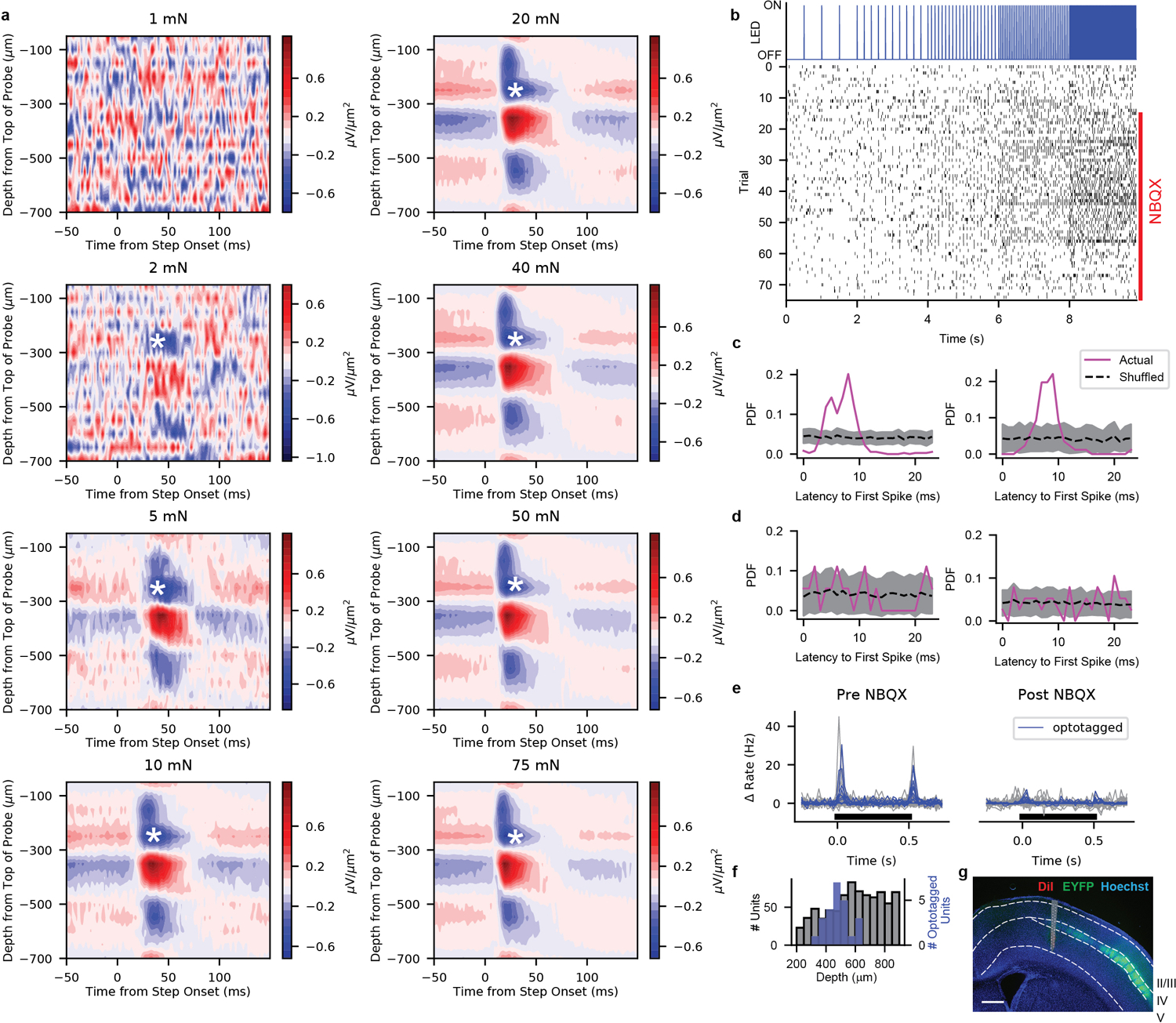
Depth Calibration and Validation for S1 Recordings **a**, Current source density (CSD) plots of an exemplar hindpaw wild-type S1 recording. Sources (red) and sinks (blue) are apparent soon after the onset of the step indentation. The depth of an early, prolonged sink (marked by an asterisk) was used to rigidly adjust the depth of the probe so that this sink was at the center of layer IV. **b**, Optotagging protocol (top) and corresponding action potential timing of an example optotagged unit (bottom) from an *Scnn1a-tg3-Cre;R26*^*LSL-ChR2*^ mouse. NBQX (5 mM) was applied to the surface of the brain to block excitatory synaptic transmission starting on trial 16. **c**, Probability distributions of the latency to the first spike after LED pulses for two optotagged units. Shaded region represents 95% confidence interval of shuffled distribution. **d**, Probability distributions of the latency to the first spike after LED pulses for two non-optotagged units. Shaded region represents 95% confidence interval of shuffled distribution. **e**, Mechanical responses to 75-mN step indentations for each unit in a recording from a *Scnn1a-tg3-Cre;R26*^*LSL-ChR2*^ mouse before (left) and after (right) application of NBQX. Optotagged units (blue) had similar mechanical response profiles to non-optotagged units (gray). **f**, Depth distribution (after CSD calibration) of all units (gray, n = 866) recorded from wild-type hindpaw S1 compared with the depth of all optotagged units (blue, n = 24 from 5 recordings from 3 mice). The majority of optotagged units were within layer IV (416.5 – 535.5 μm deep). **g**, Typical location of an electrode array in hindpaw S1 superimposed upon post hoc histology of a mouse expressing ChR2-EYFP in layer IV neurons (Scnn1a-tg3-Cre;R26^LSL-ChR2-EYFP^). The probe was coated in DiI prior to recording. Scale bar, 500 μm.

We classified the laminar location of individual cortical units using the location of the spike waveforms on the probe and by comparing this location with physiological and anatomical indicators of cortical layer. We established the center of layer IV as the location of an early sink in the local field potential (LFP) current source density plot examined at the onset of skin indentation (Extended Data Fig. 2a). Voltage waveforms were low-pass filtered at 250-Hz with an 8-pole Butterworth filter to produce LFP waveforms. Current-source densities (CSDs) were calculated by taking the 2^nd^ derivative of this signal across laminar locations on the probe. The depth of each unit was determined by the center of mass for the action potential waveform across the electrodes. This depth was rigidly corrected so that electrode sites at the center of layer IV would be 476 μm below the surface. The corrected depth of each unit allowed us to classify units to cortical layers according to the following layer depths, which were measured from post-hoc brain slices:

Layer II/III: 119 – 416.5 μm
Layer IV: 416.5 – 535.5 μm
Layer V: 535.5 – 952 μm
Layer VI: Deeper than 952 μm

We validated this classification by optotagging layer IV excitatory neurons in recordings from cortices of *Scnn1a-tg3-Cre;R26*^*LSL-ChR2-EYFP*^ mice using a 32-channel optrode (Neuronexus A1×32-Poly2-5mm-50s-177-OA32LP). The 105-μm core/125-μm outer diameter, 0.22 numerical aperture, flat-cleaved optical fiber rested atop the cortical surface (positioned 1100 μm above the tip of the electrode). Brief (2 to 10 ms) pulses of light generated by a 470-nm LED (Thorlab M470F3) were delivered through the fiber in a series of increasing frequencies (ranging from 2 to 40 Hz). Total light power measured from the optrode fiber was 1.0 mW. The optical stimulation was delivered before and after mechanical protocols. Units that reliably responded to these pulses with short latencies (< 10 ms) were considered optotagged. At the end of the experiment, the bath solution was removed and replaced with 40 μl of 5 mM NBQX (in 50% DMSO and 50% extracellular saline). This greatly attenuated S1 responses to mechanical stimulation but all optotagged units continued to respond to the optical stimulation. In fact, the addition of NBQX revealed additional units that responded to light, suggesting that polysynaptic inhibition may prevent direct optical activation from generating action potentials in some ChR2-expressing layer IV neurons.

We classified cortical units as regular spiking (RS; largely excitatory neurons) or fast spiking (FS; largely parvalbumin-expressing inhibitory interneurons) based on spike waveform trough-to-peak times^41,42^. Consistent with previous measurements in mouse sensory cortices^41^, this waveform feature exhibited a bimodal distribution. RS units were designated as those with a trough-to-peak time > 0.55 ms and FS units were designated as those with a trough-to-peak time ≤ 0.55 ms.

### Movement Subtraction

To isolate passive tactile responses, the ventral aspect of the animal was illuminated with an array of 850-nm LEDs and we used video (10 Hz frame rate; FLIR BFS-U3–13Y3M-C; SpinView version 1.1.0.43) to detect and omit time periods in which the animal moved its stimulated paw. A square region of interest proximal to the stimulation site was binarized (Otsu thresholding) and the sum of the difference between adjacent frames was calculated. If the first derivative of this sum exceeded a threshold of three standard deviations between 0.25 s prior to and 0.25 s after the step indentation, the entire step was excluded from subsequent analyses.

### Analysis of Spatial Information and Receptive Fields

Receptive fields were measured by applying a series of 16 0.5-s indentation steps alternating between intensities of 2 mN and 10 mN to 36 locations in a 5 × 5 mm grid or 25 locations in a 4 × 4 mm grid for hindpaw and forepaw stimulation, respectively. For DKOs and their littermate controls, we measured RFs with 40 mN indentation steps applied to 36 locations in a 5 × 5 mm grid. The stimulation location was randomized and repeated twice so that a total of 16 repetitions of each step indentation were applied at each location.

Spatial information was quantified as the mutual information^43^ between neural activity (in 10-ms sliding peri-stimulus windows) and the stimulus location using the information breakdown toolbox^44^. There was no detectable spatial information in response to 2-mN indentations, so all analyses focused on the 10-mN indentations. RF area was estimated by first quantifying, separately for each location, the mutual information between the presence or absence of a stimulus and mean neural activity in a 50-ms window just after the onset of the step indentation as well as a 50-ms window prior to the step indentation. Then the fraction of stimulus locations where there was significant mutual information between neural activity and stimulus presence (p < 0.05, permutation test, with the null-hypothesis distribution obtained by randomly permuting within-trial stimulus presence 1000 times) was multiplied by the probed area to calculate the RF area. Mutual information quantifies the selectivity to each specific location without making assumptions about the response tuning functions of the neurons and without making assumptions about contiguity of selectivity of responses. Thus, this information theoretic measure of RF size is free of assumptions about both the shape of tuning at each individual location and about the spatial shape of tuning across locations. Sampling bias was corrected in all information measures by subtracting out the analytical estimation of the bias^45,46^. For obtaining even more conservative estimates, the sampling bias of the spatial information metric was further corrected by subtracting the amount of spatial information observed during the baseline period from all information values. There was no statistically significant spatial information during this time period (determined by comparing to information calculated from 1000 iterations of shuffled stimulus locations). Information values that were overcorrected for sampling bias (value less than 0 bits) were set to 0 bits.

RF sizes for Aβ LTMRs were calculated by multiplying the fraction of responsive sites by the skin area that was stimulated.

### Analysis of Intensity-Response Relationships

Only recordings where the intensity series was applied within 2 mm of the peak multiunit RF region (≥ 75% of the maximum multiunit response) were included for analysis of intensity-response relationships. We quantified sensitivity and response magnitude in three ways. First, we determined the fraction of units that responded to each force step. A unit was determined to be responsive if it produced |z-scored firing rate| ≥ 3 between 10 and 50 ms after the onset or offset of the step indentation. Second, we fit the intensity-response relationships with a saturating exponential, $R\;=\;1\;–\;e^{-I/I_{0}}$, where *R* is the peak-normalized mean response measured in the same 10 to 50 ms window, *I* is the intensity, and *I*_*0*_ is the fit parameter that represents sensitivity. Only units with a sum of the absolute value of residuals less than 1.2 were included. Third, we quantified the maximum response (in Hz) within a 10 to 50 ms window after the onset or offset of all step indentations.

Response durations at the onset and offset of the step indentation were calculated by determining the number of consecutive 20-ms bins that exceeded a threshold of two standard deviations.

**Fig. 1:**
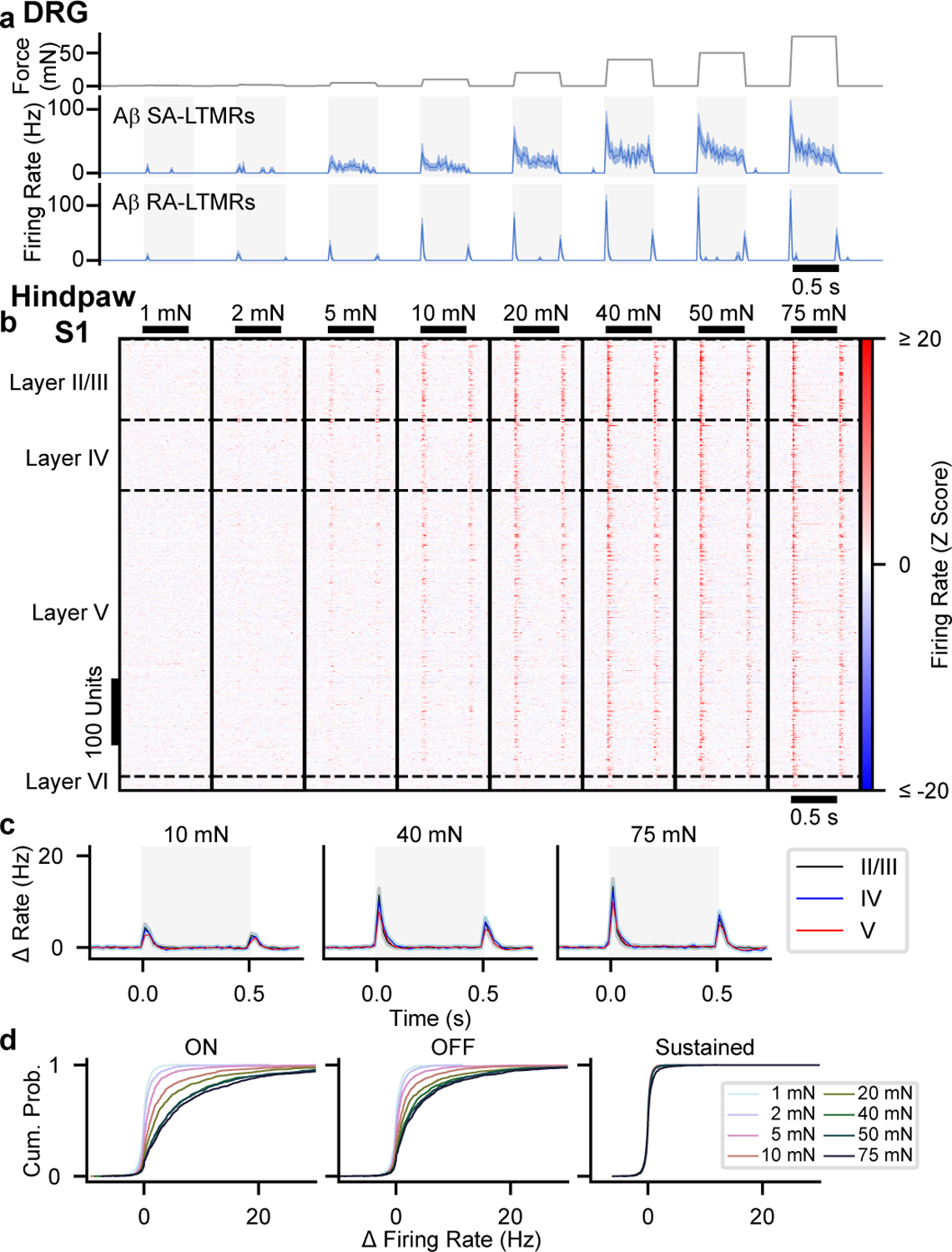
Hindpaw S1 Responses to Step Indentations **a**, *In vivo* recordings from Lumbar DRGs of anesthetized mice. Mean (± s.e.m.) firing rate responses of Aβ SA-LTMRs (top, n=11 neurons) and Aβ RA-LTMRs (bottom, n=13 neurons) to 1–75 mN indentations applied to glabrous skin. **b**, Step Indentation responses of 678 RS units. **c**, Grand mean firing rate (± s.e.m) for RS units from each layer. Shaded region indicates timing of step indentation. **d**, Cumulative distribution of baseline-subtracted firing-rate response to each step intensity for onset, offset, and sustained periods.

To compare the responses of Aβ LTMR subtypes and S1, we employed a linear model similar to that applied to macaque S1^8^. Baseline-subtracted peristimulus histograms (PSTHs; 20-ms bins) of each cortical unit were fit by the weighted sum of Aβ RA-LTMR and Aβ SA-LTMR PSTHs measured in response to the same step indentation (Fig. 1a):

$$R_{S1}=\beta_{SA}\times \;R_{SA}+\beta_{RA}\times \;R_{RA}$$

where *R*_*S1*_ is the cortical firing rate PSTH (in Hz), *R*_*SA*_ and *R*_*RA*_ are the mean PSTHs (in Hz) of the Aβ SA-LTMRs and Aβ RA-LTMRs, respectively, shifted by one 20-ms bin to account for the latency between the DRG and cortex. The y-intercept was set to 0 due to the baseline subtraction of the cortical PSTH. Only units with a significant response during any phase of the response as well as a statistically significant R^2^ value (p < 0.05; permutation test) were included in the analyses.

### *In vivo* DRG Electrophysiology

Recordings were made from the DRG using the same preparation as previously described^11,14^ and a subset of the data presented here originated from previously published recordings^14^. Briefly, anesthesia was induced with urethane (1 g/kg body weight) and maintained using 1–2% isoflurane. The L4 DRG was exposed via a dorsal incision and laminectomy. The exposed DRG was immersed in external solution containing (in mM) 140 NaCl, 3.1 KCl, 0.5 KH_2_PO_4_, 6 glucose, 1.2 CaCl_2_, 1.2 MgSO_4_ (pH adjusted to 7.4 with NaOH) and the same solution was used to fill glass pipettes with a 20–30 μm tip diameter. Fluorescent cell bodies that were labeled with genetic reporters and/or dye-conjugated CTB (ThermoFisher C34776 or C34775) were targeted for loose-seal cell-attached recordings. Extracellular action potentials were measured using a Multiclamp 700A amplifier (Axon Instruments) operated in the voltage clamp configuration. The pipette voltage was set so that no current was flowing through the amplifier at baseline. Electrophysiological data were digitized at 40 kHz with a Digidata 1550a (Molecular Devices), low-pass filtered at 10 kHz (four-pole Bessel filter), and acquired using pClamp (Molecular Devices, Version 10).

Force controlled indentations were delivered via a probe attached to the arm of an indenter (Model 300 C-I, Aurora Scientific) that was mounted on two linear motorized stages (MTS25/M-Z8E, Thorlabs) that were used to control the position of the indenter. Low-pass filtered (15-ms boxcar) force steps and sinusoidal force stimuli were synthesized in Matlab 2017b (Mathworks, Natick, MA) and a National Instruments system (NI USB 6259). Force stimuli were applied atop the minimal holding force required to keep the indenter probe in contact with the skin.

### Optical Skin Stimulation and Analysis

For optical stimulation of Aβ LTMRs expressing ReaChR^47^, pulses of light were generated every 150 ms by a 300 mW, 445 nm laser (CST-H-445–300, Ultralasers, Inc.). A total of 5000 light pulses were directed to the paw through two galvanometer scan mirrors (GVS201, Thorlabs) and an Fθ lens (FTH100–1064, Thorlabs), which focused the light to a 79 μm diameter spot (measured with a beam profiler [BP209-VIS/M, Thorlabs]). The intensity was modulated by inserting neutral density filters into the light path between the laser and the scan mirrors. Pulses were 0.3 ms in duration and the location of each pulse was randomized yet confined to an 8 × 8 mm area that encompassed the entire glabrous skin region of the paw. The location and timing of the light pulses were controlled using voltage signals generated with Matlab (2017a, Mathworks) and a National Instruments system (NI USB 6259).

Z-scored firing rate was calculated in 1-ms bins using the baseline mean and standard deviation in the 10 ms preceding each laser pulse. Units were determined to be responsive to the optical stimuli if the absolute value of the Z-scored firing rate exceeded 2.58 (99% confidence interval) between 5 and 25 ms after the laser pulse. To calculate number of evoked spikes per stimulus, we calculated binned (0.25 mm × 0.25 mm × 8 ms) spatiotemporal responses to laser pulses. We then filtered the responses with a 2-dimensional spatial gaussian (0.5 mm width) to determine the spatial location with the highest response. We calculated the number of evoked spikes per optical pulse for all pulses within 450 μm of this most responsive location. We estimated that ~4,000 S1 neurons share a mechanical receptive field by multiplying the neuron density of mouse sensorimotor cortical areas^48^ by the volume of forepaw S1 corresponding to one cytochrome oxidase-dense domain^49^.

In the DRG, the latency between the optical stimulus was calculated as the median latency of responses to pulses applied within 450 μm of the mechanical RF center. For optically responsive VPL and S1 units, we compared the distribution of first spike latencies after each optical pulse to shuffled distributions (100 shuffles) in which the timing of the optical pulses was randomized. Latencies were determined to be the time at which the actual distribution exceeded the 95% confidence interval of the shuffled distributions.

### K-Means Clustering

Clustering of VPL units was performed on the first three principal components (accounting for 76% of the variance) on the z-scored responses to 75-mN indentations. k = 4 was chosen because it was the maximum value of k that clustered multiple units from both the *TrkC::ReaChR* and *TrkB::ReaChR* lines into all clusters. PCA and k-means clustering was implemented with the scikit-learn python package.

### Histology

Mice were euthanized by inhalation of 100% CO_2_. Paws were removed and fixed for 24 h in Zamboni fixative at 4 °C. Paws were rinsed in phosphate-buffered saline (PBS) four times for 30 minutes each. The glabrous skin was dissected away from the hindpaws and forepaws and were then cryoprotected in 30% sucrose (in PBS) at 4 °C overnight. After freezing in a dry ice and ethanol bath, cryosections (25 to 35 μm thick) were mounted directly to slides.

Sections were rehydrated with PBS, then permeabilized with two washes of 0.1% Triton-X 100 in PBS (PBST), and then blocked in 5% normal goat serum in PBST for 1 hr at room temperature. Primary antibodies were diluted in 5% normal goat serum in PBST and tissues were incubated in a humidified chamber overnight at 4 °C. Primary antibodies included chicken polyclonal anti-GFP (Aves Labs AB_2307313; 1:500), chicken polyclonal anti-NFH (Aves Labs AB_2313552; 1:500), rabbit polyclonal anti-S100 beta (ThermoFisher 15146–1-AP; 1:300), and rat monoclonal anti-Troma1 (DSHB AB_531826; 1:200). The tissue was washed four times with PBST (at least 5 minutes each) before incubation with secondary antibodies (all diluted 1:500 in 5% normal goat serum in PBST) for 1–2 hr at room temperature. Secondary antibodies included goat anti-chicken conjugated to Alexa 488 (Thermofisher A-11039), goat anti-rat conjugated to Alexa 546 (Thermofisher A-11081), goat anti-rabbit conjugated to Alexa 546 (Thermofisher A-11035), goat anti-rabbit conjugated to Alexa 647 (Thermofisher A-21245), and goat anti-rat conjugated to Alexa 647 (Thermofisher A-21247). The tissue was then washed four times (at least 5 minutes each) with PBST, one of which contained Hoechst 33258 (Millipore Sigma 94403) diluted 1:2000, and then washed twice with PBS and imaged using a confocal microscope.

To quantify the density of Merkel cells and Meissner corpuscles in DKOs and their controls, the total number of Merkel cells and corpuscles in all sections was divided by the area of the epidermal border within pedal pads (estimated by tracing the border between the epidermis and dermis to determine border length using FIJI and multiplying this length by the section thickness).

### Data Analysis and Statistics

Data were analyzed in Matlab (versions 2017a and 2017b) and python (version 3.7.7) using the following packages (versions in parentheses): conda (4.8.5), matplotlib (3.3.1), numpy (1.18.5), pims (0.5), pyabf (2.2.6), scipy (1.5.2), scikit-image (0.16.2), scikit-learn (0.23.2), and seaborn (0.11.0). All statistical tests were nonparametric and performed as two-way comparisons. Pearson’s r values were calculated using the least-squares method for linear regression.

## Extended Data

**Extended Data Fig. 3:**
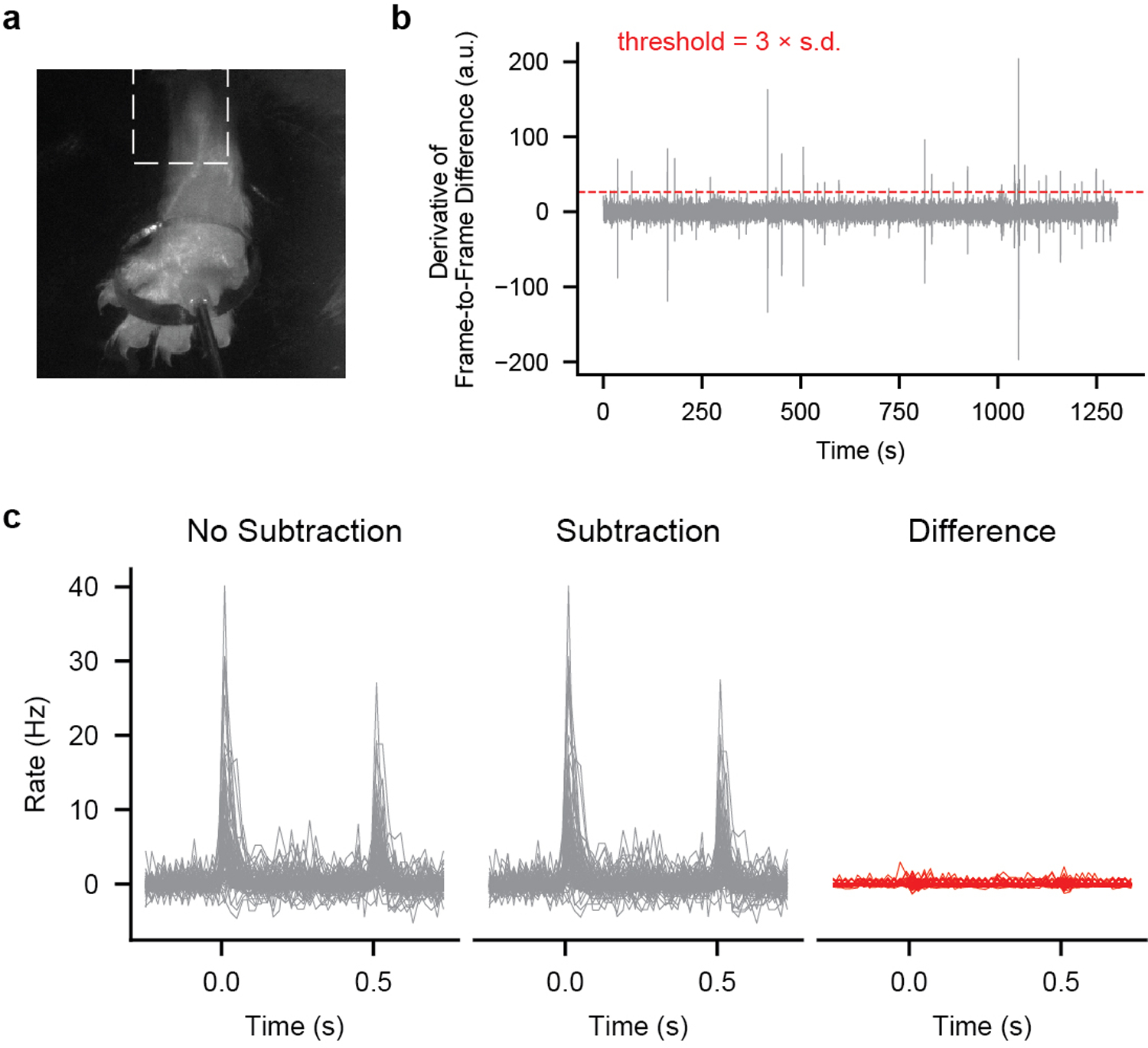
Movement Subtraction **a**, Frame of video (taken at 10 Hz) of paw during stimulation. Dashed box outlines region of interest (ROI) used for movement analysis. The ROI was binarized using Otsu thresholding and the difference from frame to frame was calculated. **b**, The first derivative of the frame to frame difference for an example recording. When this derivative exceeded three standard deviations from 0.25 s before the step to 0.25 s after the step, the entire step was excluded from subsequent analyses. **c**, Firing rate histograms in response to 75-mN step indentations (from 0 to 0.5 s) without (left) and with (middle) subtraction from the recording in b. The difference is shown on the right.

**Extended Data Fig. 4:**
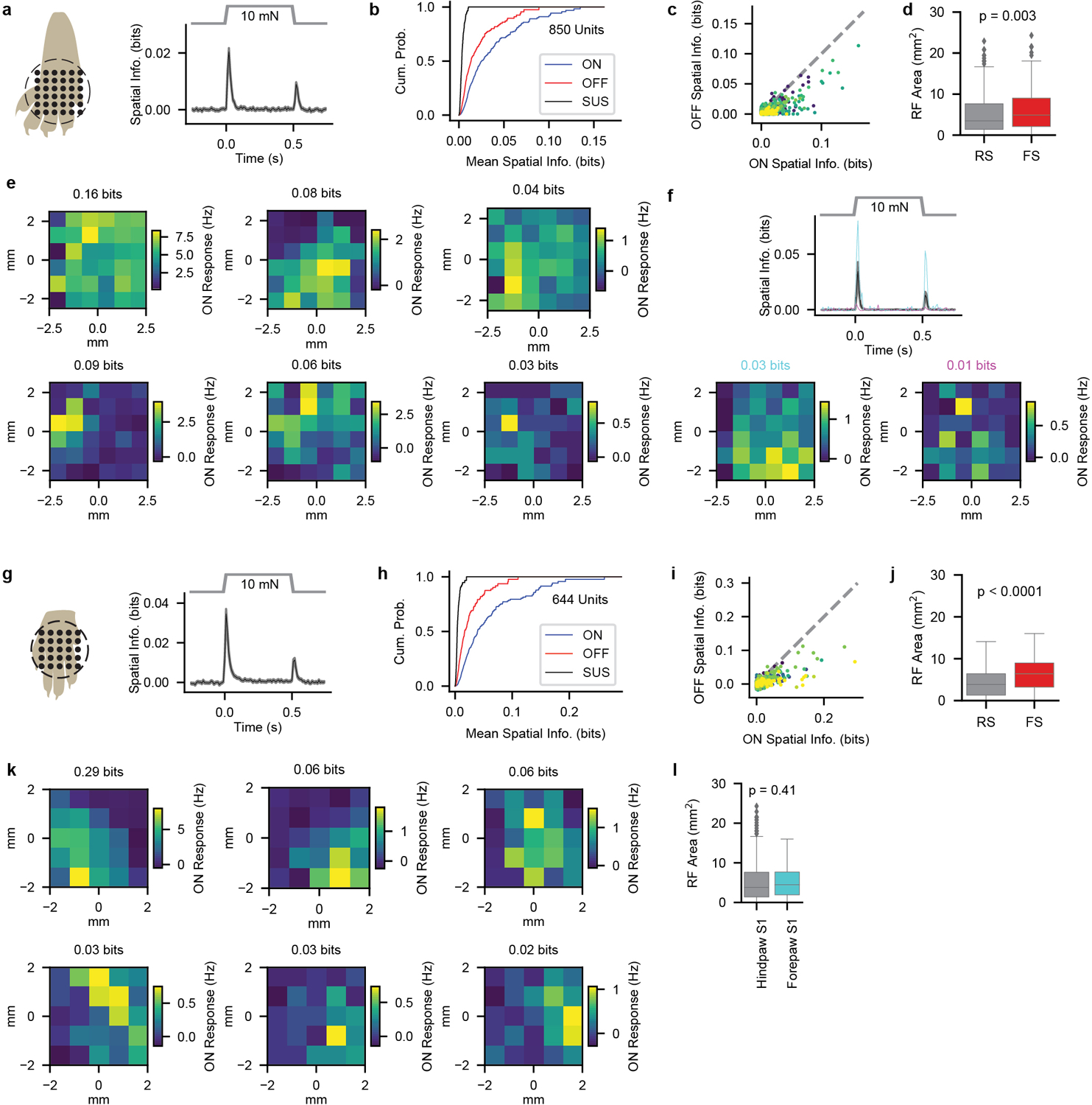
Receptive Fields and Spatial Information of Units in Hindpaw S1 and Forepaw S1 **a**, Left: stimulus locations used to probe receptive fields with 10-mN step indentations for hindpaw S1. The grid size is 5 × 5 mm. Right: Mean (± s.e.m.) spatial information for all hindpaw S1 units during the course of the 10-mN step indentation. **b**, Distribution of spatial information across all hindpaw S1 units at the onset (ON; 20–70 ms after step onset), sustained (SUS; 250–500 ms after step onset), and offset (OFF; 20–70 ms after step offset) portions of the 10-mN step indentation. **c**, A correlation (Pearson r = 0.79, p = 1.4 × 10^−179^) was apparent between the amount of spatial information at the onset and offset of the step indentation, but the amount of spatial information at the onset was reliably greater than that at the offset. Colors represent units from different recordings. Gray dashed line is the unity line. **d**, RF sizes of FS hindpaw S1 units (n=201 units) were larger than those of RS hindpaw S1 units (n=649 units). Two-sided Mann-Whitney U = 56,703, p = 1.6 × 10^−10^. Box plot element definitions: center line, median; box limits, upper and lower quartiles; whiskers, 1.5× interquartile range; points, outliers. **e**, Example RFs for units with varying degrees of spatial information at the onset. The mean spatial information over the 20–70 ms after the onset of the step indentation is displayed above the heatmap for each unit. The magnitude of the response (rather than the size of the RF) appeared to account for most of the differences in spatial information. **f**, Top: Mean (± s.e.m.) spatial information for all units in a recording from an *Scnn1a-tg3-Cre;R26*^*LSL-ChR2*^ mouse. The spatial information for two optotagged layer IV units (cyan and magenta) are overlaid. Bottom: Example RFs for these two optotagged units are qualitatively similar to those displayed in e. **g**, As in a, for forepaw S1. Stimuli were applied to 16 locations in a 4 × 4 mm grid. Thus, the spatial information calculated from forepaw S1 recordings is not directly comparable to that calculated from hindpaw S1 recordings. **h**, As in b, for forepaw S1. **i**, As in c, for forepaw S1. (Pearson r = 0.71, p = 1.2 × 10^−99^). **j**, As in d, for forepaw S1 (n=435 RS and FS units, respectively;Two-sided Mann-Whitney U = 31,348). **k**, As in e, for forepaw S1. **l**, Receptive field sizes for hindpaw (n = 850 units from 12 recordings in 8 mice) and forepaw S1 (n = 677 units from 9 recordings in 4 mice) units. Two-sided Mann-Whitney U = 266932. Box plot element definitions as in d.

**Extended Data Fig. 5:**
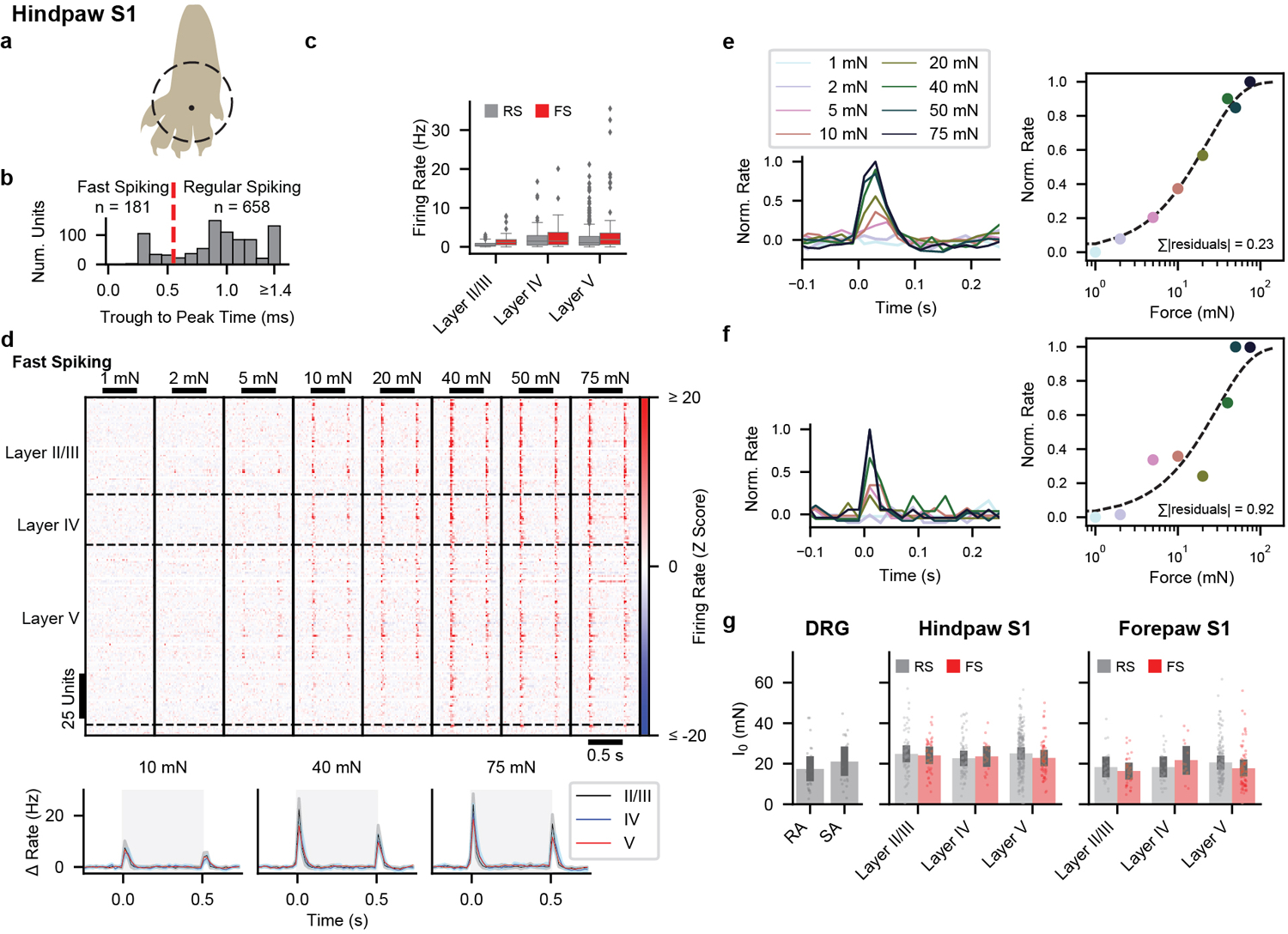
Hindpaw S1 FS Responses and Sensitivity Measurements using Fits to Saturating Exponential **a**, The hindpaw was tethered over a 7.6-mm diameter circular aperture through which step indentations were applied to the glabrous skin of the forepaw. **b**, Distribution of trough-to-peak times of action potential waveforms for hindpaw S1 units. The dashed red line demarcates the threshold (0.55 ms) used for classifying RS from FS units. **c**, Baseline firing rate for hindpaw S1 RS (n = 658 units from 12 recordings in 7 mice) and FS units (n = 181 units) from layers II/III, IV and V. Box plot element definitions: center line, median; box limits, upper and lower quartiles; whiskers, 1.5× interquartile range; points, outliers. **d**, Top: Heatmaps of the Z-scored firing rate for 181 FS units. Bottom: Grand mean firing rate (± s.e.m) for hindpaw S1 FS units from each layer. Shaded region indicates timing of step indentation. **e**, Left: Peak-normalized, baseline-subtracted firing rate at the onset of the step indentation for each force for an example hindpaw S1 RS unit. The step indentation begins at 0 s. Right: Fit of the intensity-response relation for this unit to a saturating exponential ($R\;=\;1\;–\;e^{-I/I_{0}}$). **f**, As in e for another example hindpaw S1 RS unit. **g**, Sensitivity as measured by the mean (± 95% confidence interval) *I*_*0*_ fit parameter for Aβ RA-LTMRs and Aβ SA-LTMRs (left) and well-fit (∑|residuals| < 1.2) RS and FS units in each layer of hindpaw (middle) and forepaw (right) S1. DRG n= 24 and 19 neurons for Aβ RA-LTMRs and Aβ SA-LTMRs, respectively. Hindpaw S1 n = 75 and 74 RS and FS units for layer II/III, respectively; n = 74 and 20 RS and FS units for layer IV, respectively; n = 224 and 62 RS and FS units for layer V, respectively. Forepaw S1 n = 24 and 35 RS and FS units for layer II/III, respectively; n= 27 and 13 RS and FS units for layer IV, respectively; n = 149 and 78 for layer V, respectively. No significant differences apparent within areas (DRG: Two-sided Mann-Whitney U = 183, p = 0.14; hindpaw S1: Kruskal-Wallis H = 7.04, p = 0.22; forepaw S1: Kruskal-Wallis H = 10.67, p = 0.06) but *I*_*0*_ differs between all DRG neurons and hindpaw S1 units (Two-sided Mann-Whitney U = 7594, p = 0.0004) and between all hindpaw S1 units and all forepaw S1 units (Two-sided Mann-Whitney U = 52,285, p = 9.6 × 10^−18^).

**Extended Data Fig. 6:**
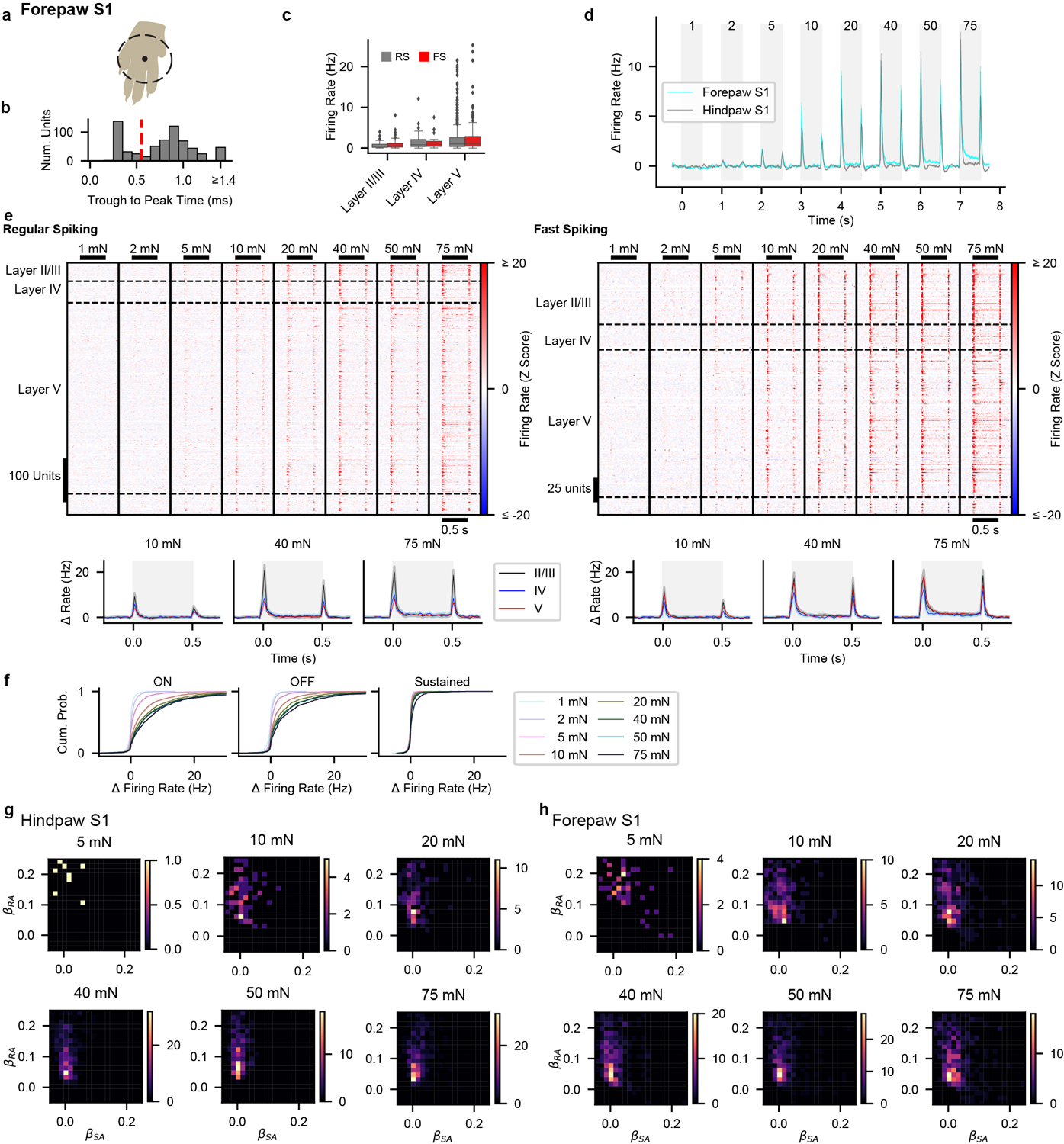
Forepaw S1 Responses to Step Indentation **a**, The forepaw was tethered over a 6.4-mm diameter circular aperture through which step indentations were applied to the glabrous skin of the forepaw. **b**, Distribution of trough-to-peak times of action potential waveforms for forepaw S1 units. The dashed red line demarcates the threshold (0.55 ms) used for classifying RS from FS units. **c**, Baseline firing rate for forepaw RS (n = 576 units from 12 recordings in 6 mice) and FS units (n = 258 units) from layers II/III, IV and V. Box plot element definitions: center line, median; box limits, upper and lower quartiles; whiskers, 1.5× interquartile range; points, outliers. **d**, Grand mean (± s.e.m.) baseline-subtracted firing rate for all forepaw S1 units (cyan; n = 834) and all hindpaw S1 units (gray; n = 866) in response to step indentations. The shaded regions represent the timing of step indentations and the numbers at the stop signify the intensity of the indentation (in mN). While a sustained response is generated within forepaw S1 to high forces, this response is dwarfed by the transients at the onset and offset of the step indentations. **e**, Top: Heatmaps of the Z-scored firing rate for forepaw S1 RS (left) and FS (right) units. Bottom: Grand mean firing rate (± s.e.m) for RS (left) and FS (right) units from each layer. Shaded region indicates timing of step indentation. **f**, Cumulative distributions of baseline-subtracted firing rate for all forepaw S1 units at each step intensity for the onset (ON; 10–50 ms after step onset), offset (OFF; 10–50 ms after step offset), sustained (SUS; 250–500 ms after step onset) periods. **g**, Sensitivity as measured by the *I*_*0*_ parameter for RS and FS units in each layer of forepaw S1 fit well (∑|residuals| < 1.2) with a saturating exponential. Number of units indicated on each bar. **h**, Density histograms of the β coefficients for the Aβ RA-LTMR and Aβ SA-LTMR profiles that best fit hindpaw S1 units at forces designated above each plot. Heatmap colors represent number of units per bin. Only units with significant R^2^ values, as determined by permutation of the LTMR response profiles, were included. **i**, As in h, for forepaw S1 units.

**Extended Data Fig. 7:**
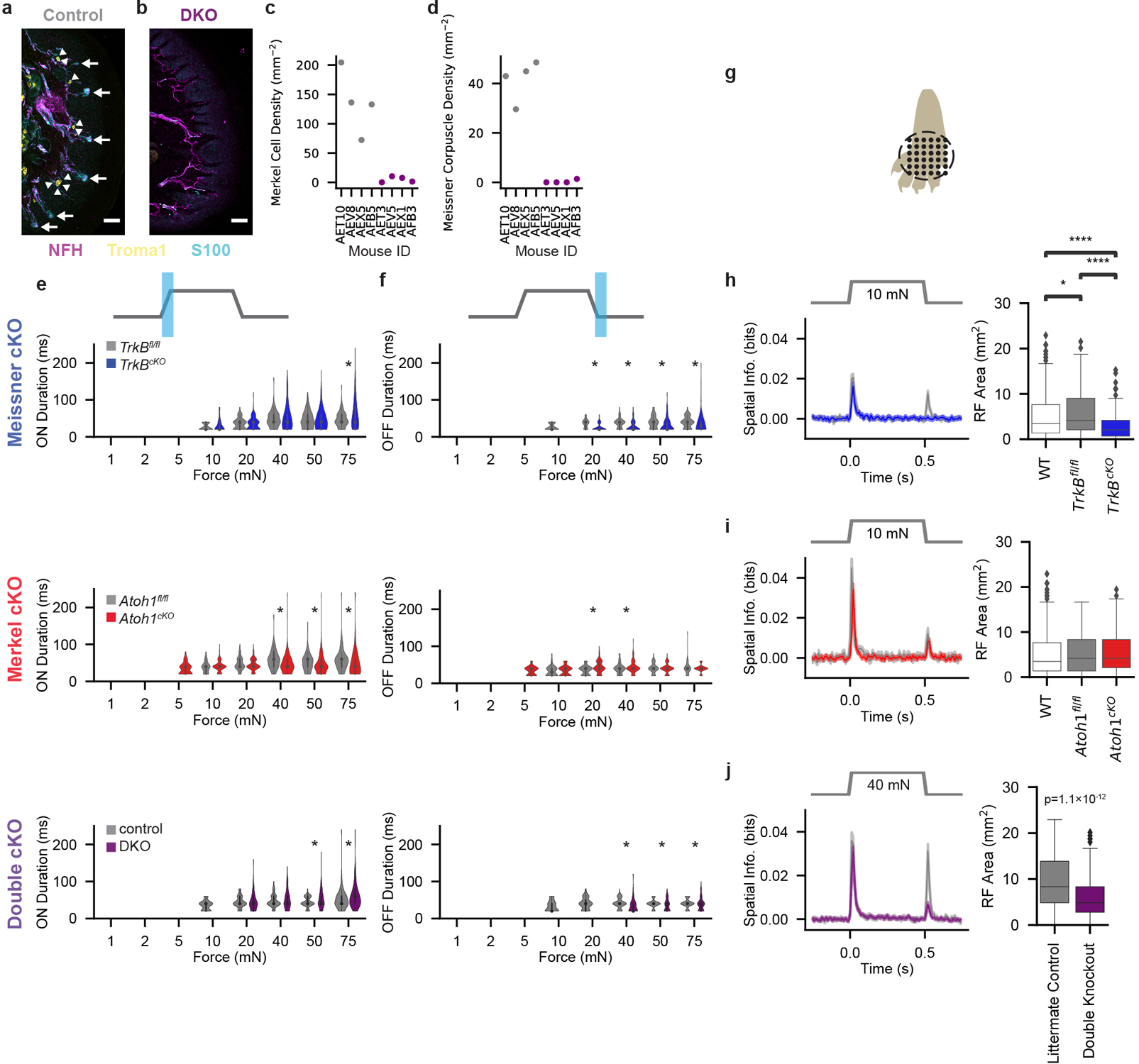
DKO Histology and S1 Receptive Fields and Response Durations in Knockout Mice **a**, Example pedal pad glabrous skin section in a littermate control (AET10) immunostained for NFH (magenta) to identify axons, Troma1 (yellow) to identify Merkel cells (indicated by arrowheads), and S100 (cyan) to identify Meissner corpuscles (indicated by arrows). Scale bar: 50 μm. Similar pattern observed in four littermate controls. **b**, Pedal pad glabrous skin section in a DKO (AEV5) immunostained for NFH (magenta), Troma1 (yellow), and S100 (cyan). No Merkel cells or Meissner corpuscles were apparent in this section. Scale bar: 50 μm. Similar pattern observed in four DKOs. **c**, Quantification of the density of Merkel cells within pedal pads for littermate controls (gray markers) and DKOs (purple markers). **d**, Quantification of the density of Meissner corpuscles within pedal pads for littermate controls (gray markers) and DKOs (purple markers). **e**, Durations of responses (violin plot shows kernel destiny estimate of underlying distribution) at the onset of step indentations (calculated by multiplying the number of consecutive bins with a Z score > 2 by the bin size [20 ms]) for hindpaw S1 units sensitive to each force in control *TrkB*^*fl/fl*^ and *TrkB*^*cKO*^ mice (top), in control *Atoh1*^*fl/fl*^ and *Atoh1*^*cKO*^ mice (middle), and in littermate controls and DKO mice (bottom). Plots shown only for forces to which at least 20 units responded at the indentation onset. *p < 0.05, Two-sided Mann-Whitney U test. **f**, As in e, for responses at the offset of step indentations. **g**, Schematic of RF measurements. 10-mN indentations were delivered to 36 locations in a 5 × 5 mm grid for wild-type animals, *TrkB*^*fl/fl*^ controls, *TrkB*^*cKO*^ mice, *Atoh1*^*fl/fl*^ controls, and *Atoh1*^*cKO*^ mice. 40-mN indentations were delivered at each location for DKOs and their littermate controls. **h**, Mean (± s.e.m.) spatial information (left; blue: *TrkB*^*cKO*^, gray: *TrkB*^*fl/fl*^) and mean (± s.e.m.) RF areas (right) for S1 units from wild-type (n = 649 units), *TrkB*^*fl/fl*^ (230 units), and *TrkB*^*cKO*^ (182 units) animals. *p < 0.05, ****p < 0.0001, Two-sided Mann-Whitney U test, Bonferroni corrections for multiple comparisons applied. **i**, As in h, for RF measurements in *Atoh1*^*fl/fl*^ (gray; n = 48 units) and *Atoh1*^*cKO*^ (red; 128 units) animals. **j**, As in h, for RF measurements (made at 40 mN) in DKOs (purple; n = 369 units) and their littermate controls (gray; 217 units). Two-sided Mann-Whitney U = 5,412. Box plot element definitions (h-j): center line, median; box limits, upper and lower quartiles; whiskers, 1.5× interquartile range; points, outliers.

**Extended Data Fig. 8:**
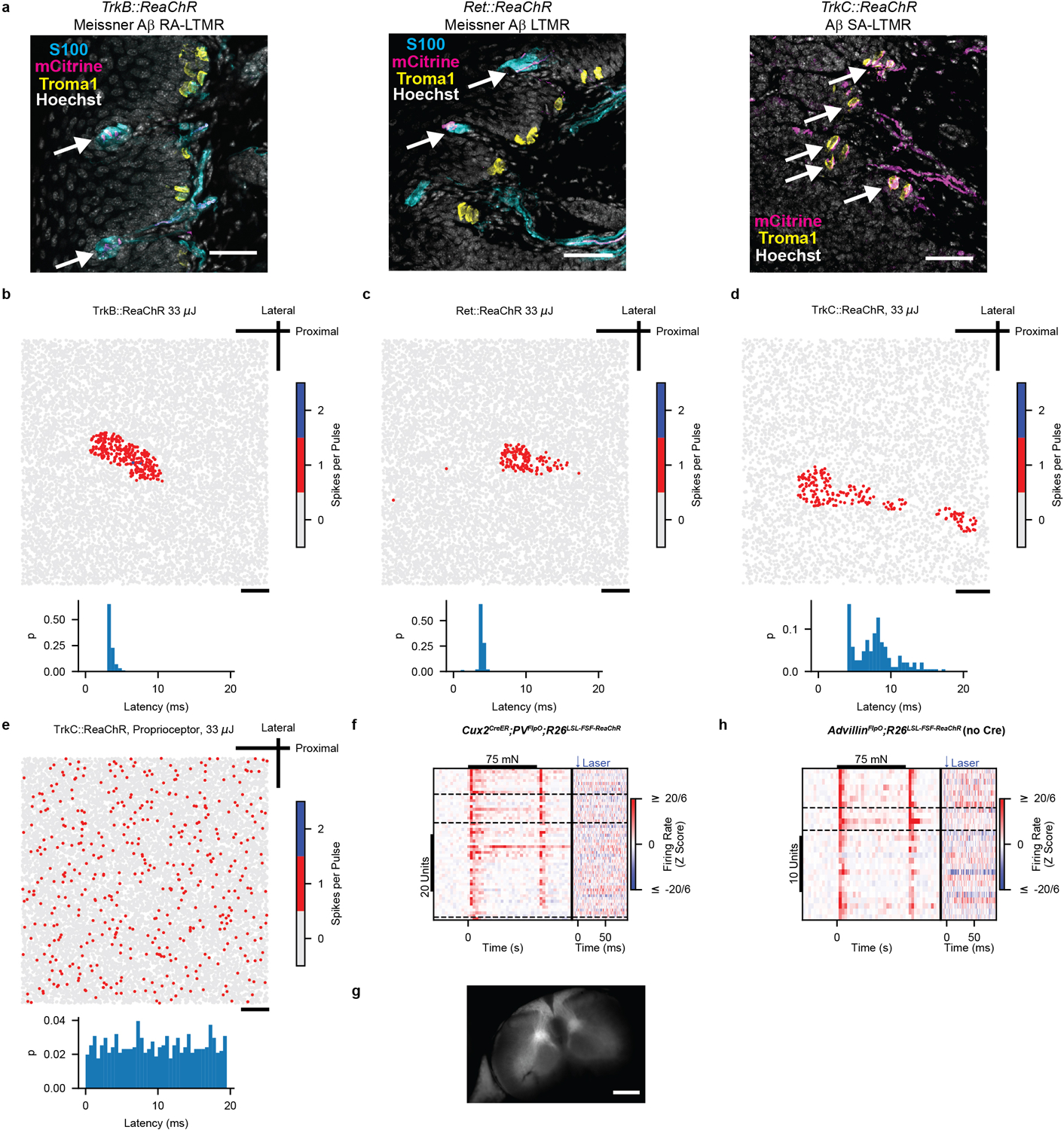
Optical Responses in Aβ LTMRs and Controls for Optogenetic Gain-of-Function Experiments **a**, Aβ LTMR subtypes selectively labeled in a *TrkB*^*CreER*^*;R26*^*LSL-ReaChR-mCitrine*^ (*TrkB::ReaChR*) mouse (left), a *Ret*^*CreER*^*;Advillin*^*FlpO*^*;R26*^*LSL-FSF-ReaChR-mCitrine*^ (*Ret::ReaChR*) mouse (middle), and a *TrkC*^*CreER*^*;R26*^*LSL-ReaChR-mCitrine*^ (*TrkC::ReaChR*) mouse (right). Arrows indicate mCitrine^+^ fibers within S100^+^ Meissner corpuscles (left and middle) or abutting Troma1^+^ Merkel cells (right). Similar patterns observed in all 7 *TrkB::ReaChR*, 8 *Ret::ReaChR*, and 12 *TrkC::ReaChR* mice. Scale bars: 40 μm. **b**, Top: 33 μJ light pulses were directed to the skin at each location indicated by a marker. A ReaChR-expressing TrkB^+^ Aβ RA-LTMR responded with, in most cases, a single action potential when the pulses were directed onto the mechanical RF of the neuron. Bottom: Histogram showing the distribution of latencies to the first spike for all locations in which an action potential was evoked by optical stimulation. Scale bar: 1 mm. **c**, As in a, for a ReaChR-expressing Ret^+^ Aβ LTMR. Scale bar: 1 mm. **d**, As in a, for a ReaChR-expressing TrkC^+^ Aβ SA-LTMR. Scale bar: 1 mm. **e**, As in a, for a ReaChR-expressing TrkC^+^ proprioceptor. This proprioceptor responded to movement of a digit. Light did not evoke action potentials, even during ongoing activity. Similar results obtained in 4 additional proprioceptors from 3 mice. Scale bar: 1 mm. **f**, Hindpaw S1 recordings from mice (n = 3) in which proprioceptors expressed ReaChR, driven intersectionally using the *Cux*^*CreER*^ and *PV*^*FlpO*^ driver lines. No responses to optical stimulation were observed in S1 despite responsivity to mechanical stimulation. Dashed lines demarcate cortical layers. **g**, Native mCitrine fluorescence in Clark’s column and the dorsal column of the cervical spinal cord of a *Cux2*^*CreER*^*;PV*^*FlpO*^*;R26*^*LSL-FSF-ReaChR-mCitrine*^ animal. Similar pattern observed in 2 additional mice. Scale bar: 500 μm. **h**, No optical responses were observed in hindpaw S1 of an *R26*^*LSL-FSF-ReaChR*^ animal lacking Cre recombinase. Dashed lines demarcate cortical layers.

**Extended Data Fig. 9:**
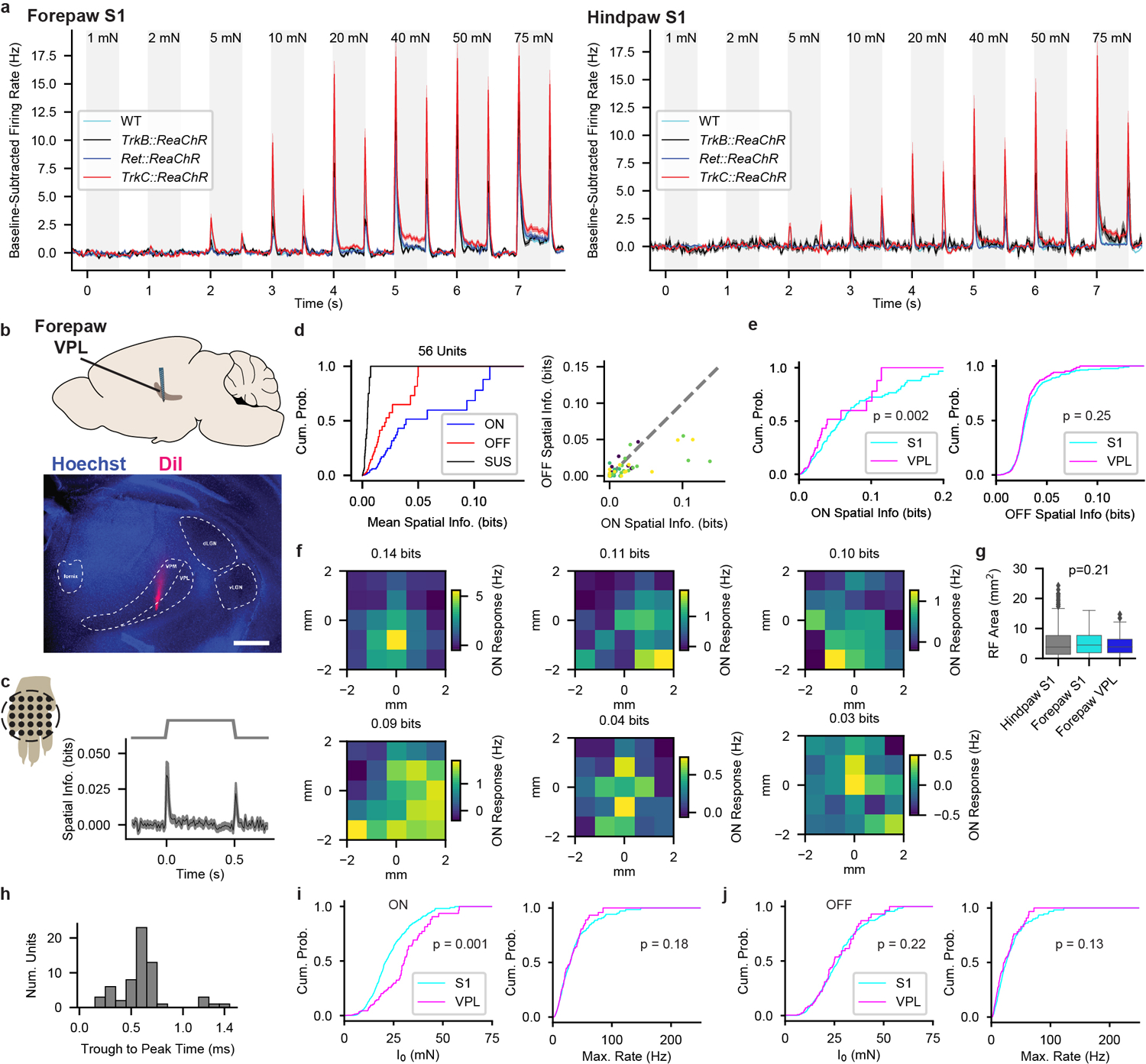
Response Profiles of S1 Units Sensitive to Selective Optogenetic Stimulation and Receptive Fields and Intensity-Response Relationships in VPL **a**, Grand mean (± s.e.m.) firing rate responses to step indentations for forepaw S1 (left) and hindpaw S1 (right) wild-type (cyan) units compared to that of units in each driver line that were responsive to optical stimulation. The response profiles are similar across all driver lines and wild-type units. Shaded regions indicate the timing of step indentations. **b**, Top: Schematic of probe position within VPL. Bottom: *Post-hoc* histology showing the location of the electrode tract (DiI, red) in relation to thalamic structures. Hoechst 33258 nuclear stain shown in blue. Scale bar: 500 μm. Similar histology observed in 3 mice. VPL: ventroposterolateral nucleus of the thalamus, VPM: ventroposteromedial nucleus of the thalamus, dLGN: dorsal lateral geniculate nucleus, vLGN: ventral lateral geniculate nucleus. **c**, Mean (± s.e.m.) spatial information of mechanically sensitive VPL units (n=56) in relation to 500-ms, 10-mN step indentations applied to 25 locations in a 4 × 4 mm grid. **d**, Left: Cumulative distribution of mean spatial information of VPL units at the onset (ON; 20–70 ms after step onset), sustained (SUS; 250–500 ms after step onset), and offset (OFF; 20–70 ms after step offset) portions of the 10-mN step indentation. Right: The amount of information at the onset and offset is correlated (Pearson r = 0.70, p = 1.3 × 10^−9^). **e**, Spatial information in VPL units (n = 56) and forepaw S1 units (n = 306) differs at the onset (left) but is indistinguishable at the offset (right) of the step indentation. Two-sided Mann-Whitney U = 6,520 and 8,071 for onset and offset comparisons, respectively. **f**, RFs of example VPL units with varying amounts of mean spatial information at the onset of the step indentation, noted above each heat map. **g**, RF area for S1 and VPL units (n = 850, 644, and 56 units for hindpaw S1, forepaw S1, and VPL, respectively). Kruskal-Wallis H = 3.12. Box plot element definitions: center line, median; box limits, upper and lower quartiles; whiskers, 1.5× interquartile range; points, outliers. **h**, Distribution of action potential waveform trough to peak times for mechanically sensitive VPL units. **i**, Sensitivity of VPL units compared to forepaw S1 units at the onset of the step indentation as assessed by *I*_*0*_ fits to saturating exponentials (left; n = 80 and 342 units for VPL and S1, respectively; U = 9,026) and maximum response firing rate (right; n = 174 and 599 units for VPL and S1, respectively; U = 51,650). Mann-Whitney U test. **j**, Sensitivity of VPL units at the offset of the step indentation as assessed by *I*_*0*_ values (left; n = 64 and 316 units for VPL and S1, respectively; U = 9,502) and maximum response firing rate (right; n = 174 and 599 units for VPL and S1, respectively; U = 40,986). Mann-Whitney U test.

**Extended Data Fig. 10:**
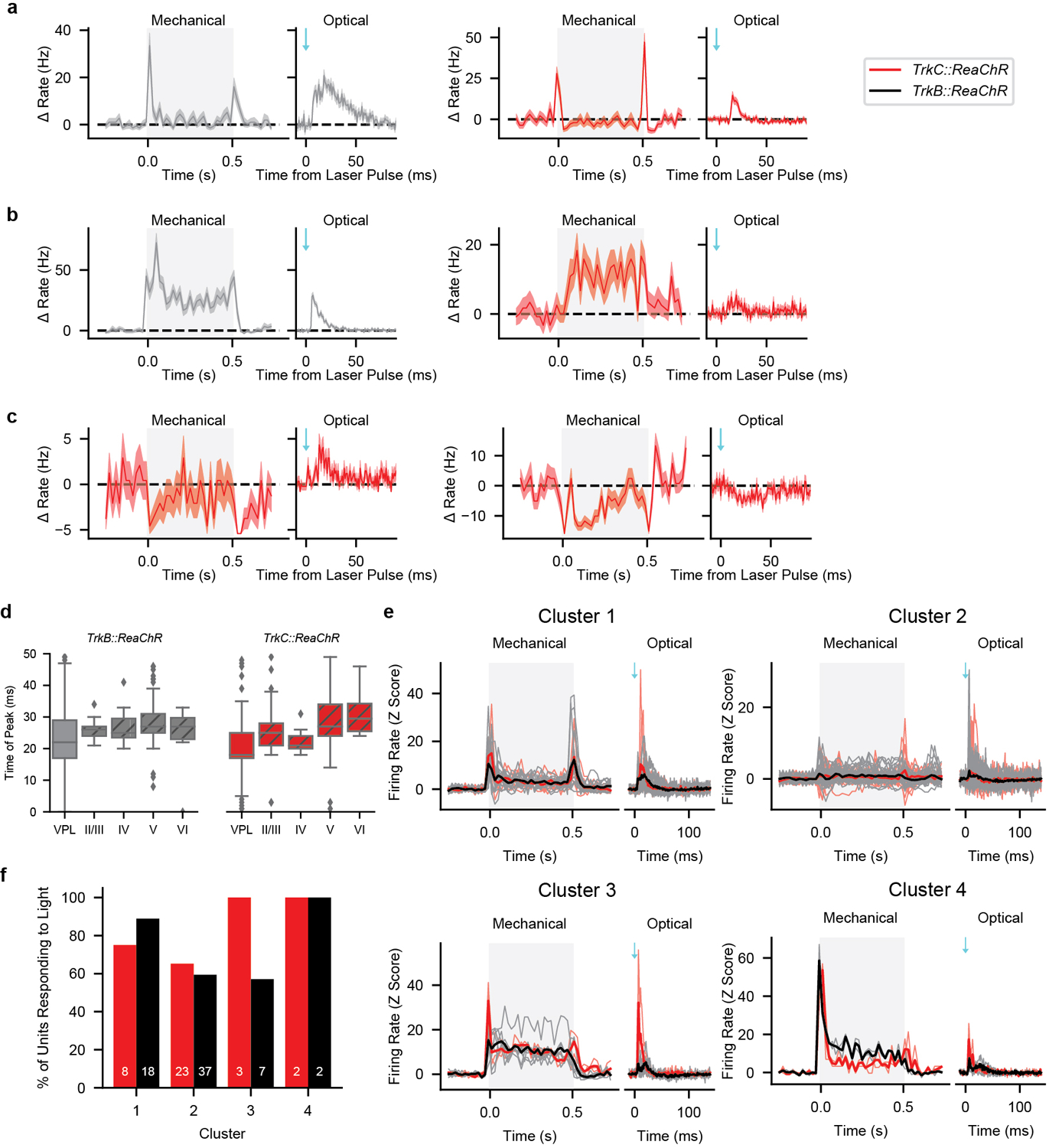
Optogenetic Activation of Aβ RA-LTMRs and Aβ SA-LTMRs Modulate Firing Rates in VPL Units with Heterogeneous Response Profiles to Mechanical Indentations **a**, Mean (± s.e.m.) firing rate of two example VPL units that respond to 75-mN step indentations by transiently increasing their firing rate at the onset and offset of the step indentation, much like typical S1 units. The unit on the left can be driven by selective optical activation of Aβ RA-LTMRs and the unit on the right can be driven by selective optical activation of Aβ SA-LTMRs. **b**, Mean (± s.e.m.) of two VPL units with prominent sustained responses to 75-mN step indentations. The unit on the left can be driven by optical activation of Aβ RA-LTMRs and the unit on the right can be driven by optical activation of Aβ SA-LTMRs. **c**, Mean (± s.e.m.) firing rate of two VPL units that respond to the 75-mN step indentation with decreases in their firing rates. The firing rate of both units can be modulated by optical activation of Aβ SA-LTMRs but the unit on the left increases its firing rate while the unit on the right decreases its firing rate. **d**, Time of peak |firing rate| relative to the laser pulse for optically sensitive units in VPL and each layer of forepaw S1 *TrkB::ReaChR* (left; U = 9,038, p = 2.6 × 10^−6^ for all VPL units compared to all forepaw S1 units, Mann-Whitney U test; n = 119, 34, 21, 152, and 10 units in VPL, layers II/III, IV, V, and VI, respectively) and *TrkC::ReaChR* (right; U = 4,828, p = 2.8 × 10^−9^ for all VPL units compared to all forepaw S1 units, Mann-Whitney U test; n = 84, 34, 39, 120, and 11 units in VPL, layers II/III, IV, V, and VI, respectively) mice. Box plot element definitions: center line, median; box limits, upper and lower quartiles; whiskers, 1.5× interquartile range; points, outliers. **e**, Each panel shows mechanical (75 mN step indentation from 0 to 0.5 s) and optical (0.3 ms pulse at 0 ms) responses of one of four clusters determined by K-means clustering of the first three principal components of the Z-scored response to 75-mN step indentations. Individual (thin lines) and mean (thick lines) Z-scored responses from units from *TrkC::ReaChR* (red) and *TrkB::ReaChR* (black) mice. Both the mechanical and optical responses generated by stimulation of mice from either genotype are similar within clusters. **f**, The majority of units from each cluster respond to selective optical activation of Aβ SA-LTMRs (*TrkC::ReaChR*, red) or Aβ RA-LTMRs (*TrkB::ReaChR*, black). Total number of units indicated on each bar.

## Figures and Tables

**Fig. 2:**
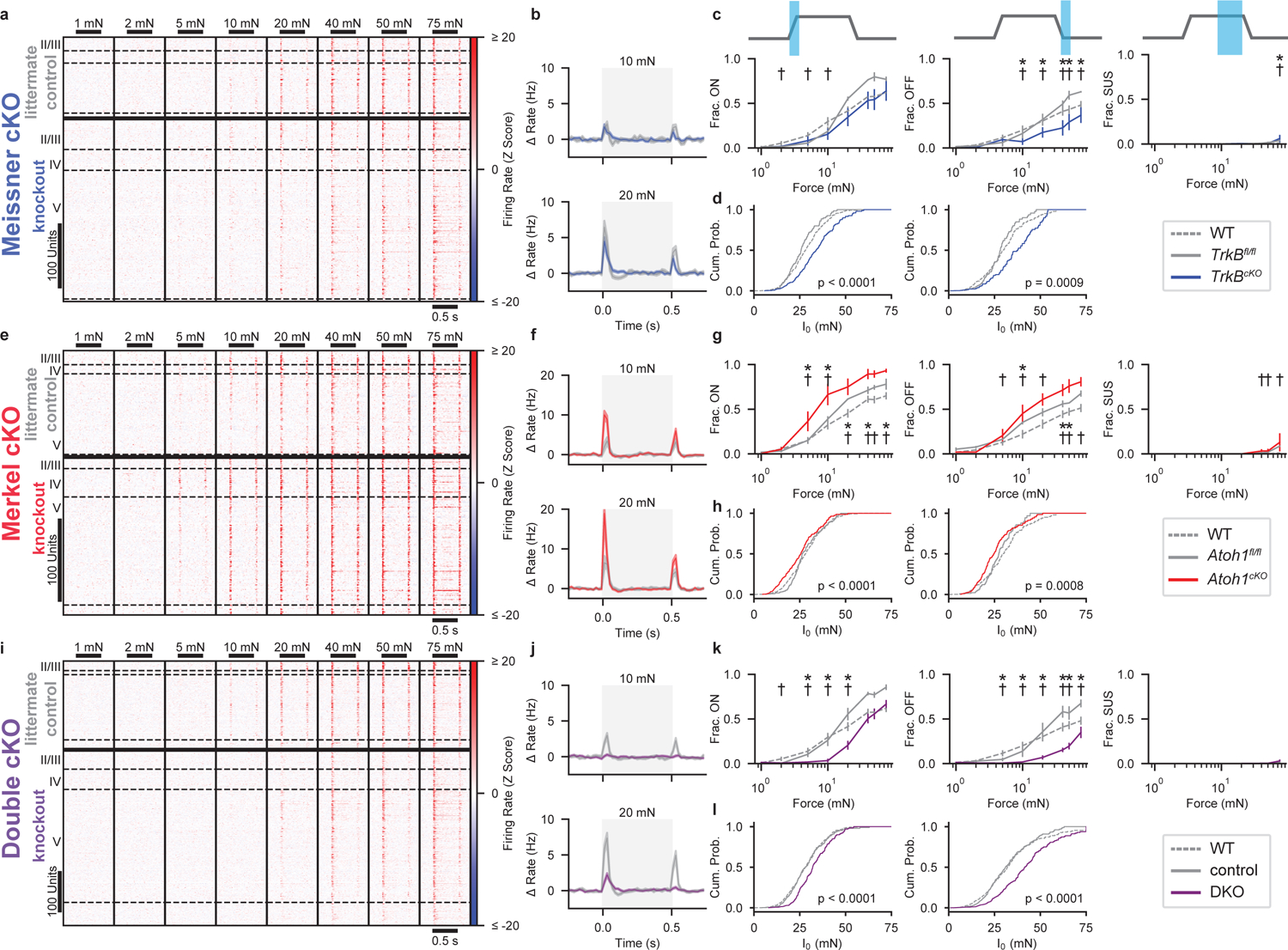
S1 in Mice Lacking Meissner Corpuscles and/or Merkel Cells Exhibits Shifted Sensitivity **a**, Indentation responses of hindpaw S1 RS units in *TrkB*^*fl/fl*^ controls (top; n=282 units, 5 recordings, 3 animals) and *Advillin*^*Cre*^*;TrkB*^*fl/fl*^ (*TrkB*^*cKO*^; bottom; n=281 units, 6 recordings, 5 animals) mice that lack Meissner corpuscles^14^. Sorted by depth. Dashed lines demarcate layers. **b**, Grand mean (± s.e.m.) of firing-rate response to indentations across control *TrkB*^*fl/fl*^ (gray) and *TrkB*^*cKO*^ (blue) RS units. **c**, Fraction (± s.e.m.) of wild-type (dashed), *TrkB*^*fl/fl*^ (gray), and *TrkB*^*cKO*^ (blue) RS units responsive at onset (left), offset (middle), or sustained (right) phases of indentations *p<0.05 for comparisons between *TrkB*^*fl/fl*^ and *TrkB*^*cKO*^ units. †p<0.05 for comparisons between wild-type and *TrkB*^*cKO*^ units, two-proportions Z test corrected for multiple comparisons. **d**, CDFs of *I*_*0*_ for onset (left) and offset (right) responses for wild-type (dashed), *TrkB*^*fl/fl*^ (gray), and *TrkB*^*cKO*^ (blue) RS units well-fit by a saturating exponential ($R\;=\;1\;–\;e^{-I/I_{0}}$). Two-sided Mann-Whitney U test. **e**, As in a, for RS units in hindpaw S1 of *Atoh1*^*fl/fl*^ controls (top; n=216 units, 3 recordings, 3 animals) and *K5-Cre;Atoh1*^*fl/fl*^ (*Atoh1*^*cKO*^; bottom; n=189 units, 4 recordings, 3 animals) mice that lack Merkel cells^18^. **f**, As in b, for *Atoh1*^*fl/fl*^ (gray) and *Atoh1*^*cKO*^ (red). **g**, As in c, for wild-type (dashed), *Atoh1*^*fl/fl*^ (gray), and *Atoh1*^*cKO*^ (red). **h**, As in d, for wild-type (dashed), *Atoh1*^*fl/fl*^ (gray), and *Atoh1*^*cKO*^ (red). **i**, As in a, for hindpaw S1 RS units of littermate control (top; n=300 units, 5 recordings, 4 animals) and *Advillin*^*Cre*^*;TrkB*^*fl/fl*^*;Atoh1*^*fl/fl*^ (DKO; bottom; n=566 units, 10 recordings, 4 animals) mice, which lack both Meissner corpuscles and Merkel cells. **j**, As in b, for littermate control (gray) and DKO (purple). **k**, As in c, for wild-type (dashed), littermate control (gray), and DKO (purple). **l**, As in d, for wild-type (dashed), littermate control (gray), and DKO (purple).

**Fig. 3:**
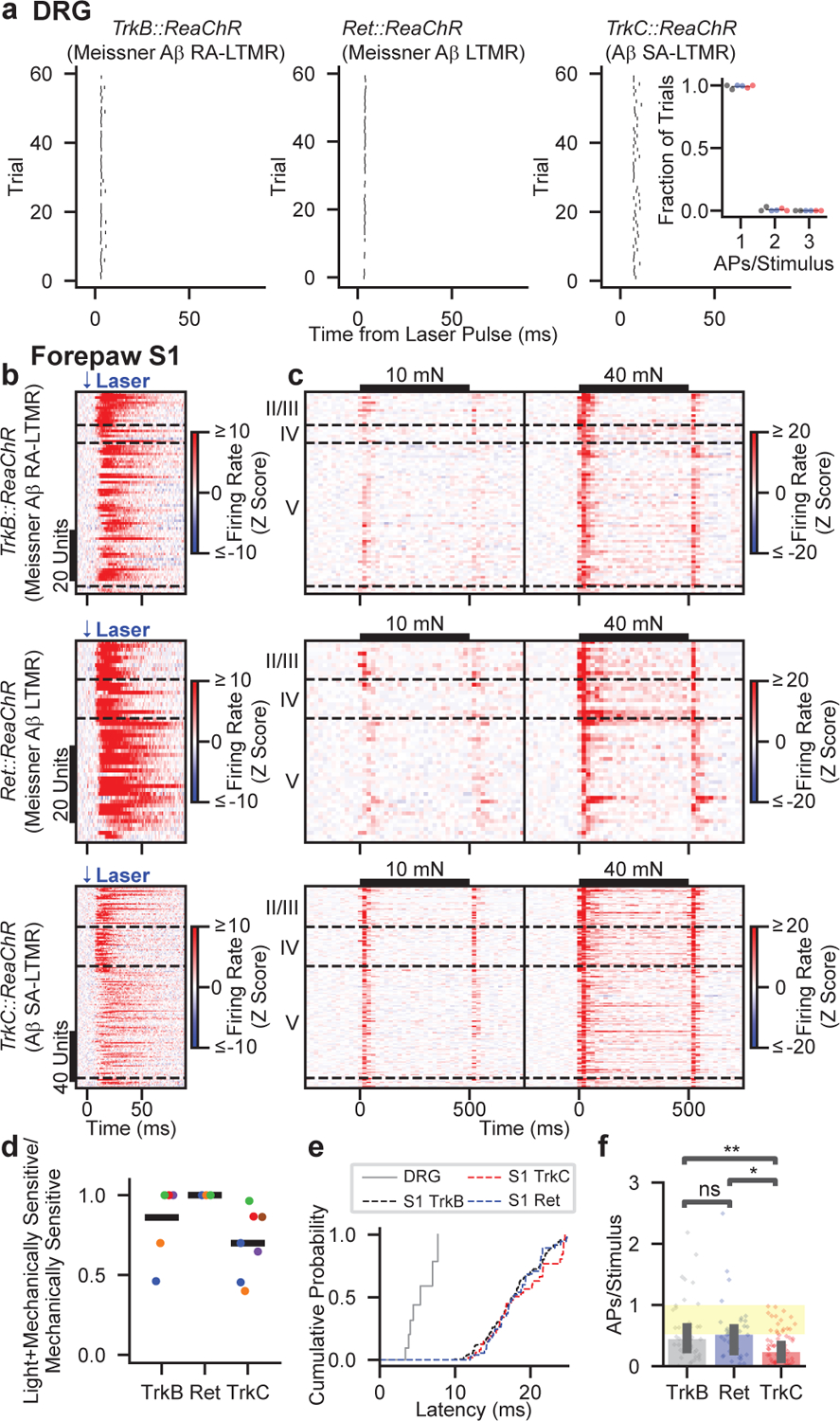
Selective Activation of Aβ LTMR Subtypes Drives the Majority of Mechanically Sensitive S1 Neurons **a**, Rasters showing action potentials (APs) evoked by optical stimulation of the mechanical RF of ReaChR-expressing Aβ LTMRs from *TrkB::ReaChR* (left; labeling Meissner corpuscle-associated Aβ RA-LTMRs), *Ret::ReaChR* (middle; labeling Meissner corpuscle-associated Aβ LTMRs), and *TrkC::ReaChR* (right; labeling Merkel cell-associated Aβ SA-LTMRs) mice. Inset: proportion of pulses that evoked one, two, or three APs. Markers represent individual LTMRs (black: *TrkB::ReaChR*; blue: *Ret::ReaChR*; red: *TrkC::ReaChR*; 6 LTMRs), bars indicate mean. **b**, Optical responses of 20-mN sensitive forepaw S1 units in *TrkB::ReaChR* (top; n=79 units, 6 recordings, 5 mice), *Ret::ReaChR* (middle; n=52 units, 5 recordings, 3 mice), and *TrkC::ReaChR* (bottom; n=159 units, 7 recordings, 6 mice) mice. Units sorted by depth. Dashed lines indicate layer boundaries. **c**, Step-indentation responses of the forepaw S1 units in c. Note the different timescale. **d**, Proportion of mechanically sensitive forepaw S1 units that respond to light. Markers represent individual recordings. Markers of the same color (within genotype) are from the same mouse. Bars represent means. **e**, First-spike latency after optical stimulation recorded from Aβ LTMRs (within DRG) and from S1 units with a latency < 25 ms. **f**, Evoked APs per stimulus for forepaw S1 units. Markers represent units, bars indicate medians, and error bars indicate 95% confidence intervals. Yellow-shaded region represents the range of values observed in Aβ LTMRs. H=13.86, p=0.00098, Kruskal-Wallis H test. Two-sided Mann-Whitney U tests corrected for multiple comparisons: **p=0.003, *p=0.016, ns p=0.94.

**Fig. 4:**
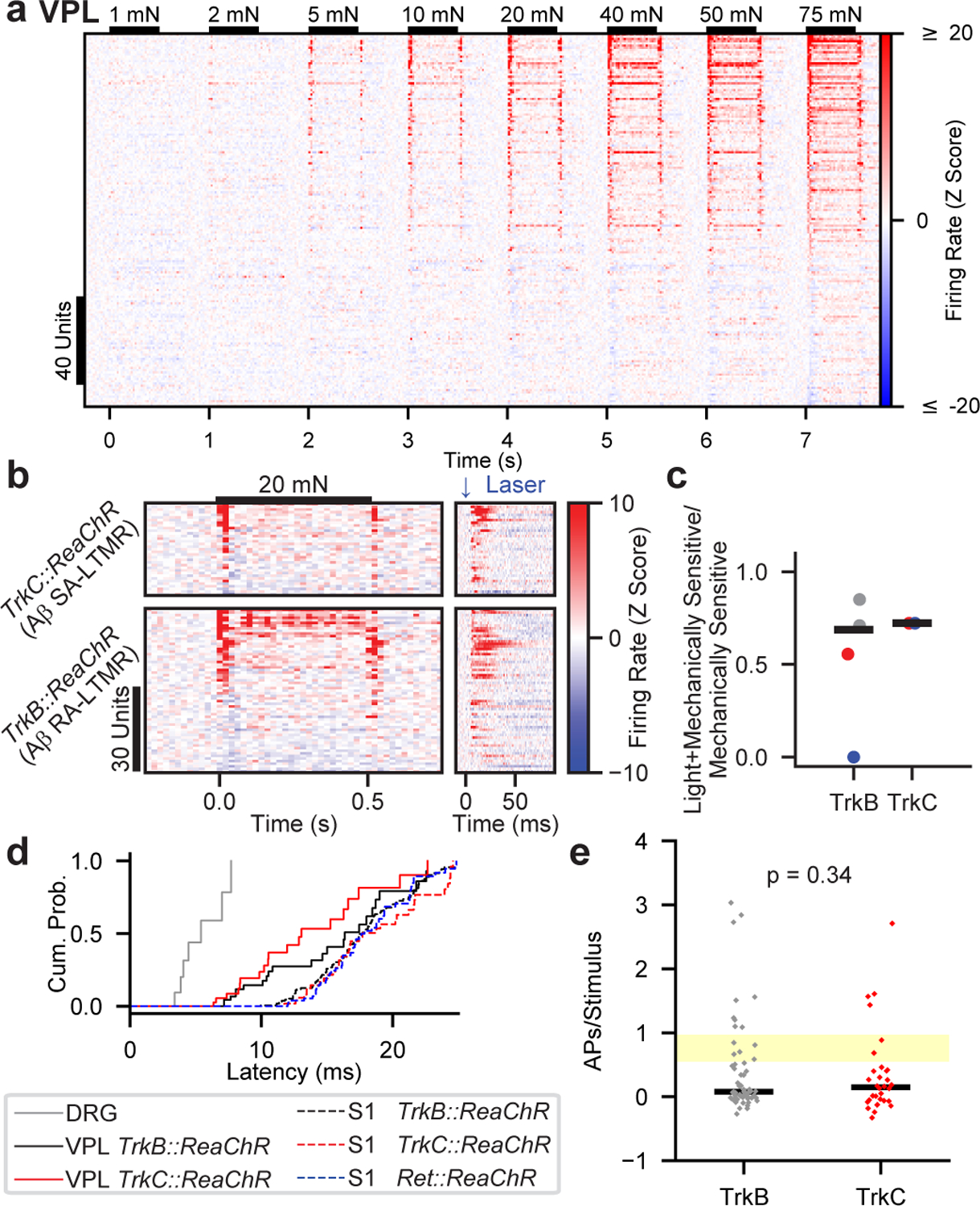
Most VPL Neurons Receive Convergent Input from Aβ RA-LTMRs and Aβ SA-LTMRs **a**, VPL unit responses to 1–75 mN step indentations. Units sorted by 75-mN ON response. **b**, Mechanical (left) and optical (right) responses in VPL units sensitive to 20-mN indentations. Optical responses driven by activation of TrkC^+^ Aβ SA-LTMRs (top; n=36 units, 2 recordings, 2 mice) or TrkB^+^ Aβ RA-LTMRs (bottom; n=64 units, 4 recordings, 3 mice). Sorted by response to indentation onset. Note the different timescales. **c**, Fraction of mechanically sensitive VPL units that respond to optical activation of Aβ RA-LTMRs (via *TrkB::ReaChR*) and Aβ SA-LTMRs (via *TrkC::ReaChR*). Markers of the same color (within genotype) represent recordings from the same mouse. Bars represent means weighted by the number of units in each recording. **d**, First-spike latencies measured in DRG, VPL, or S1 after optical stimulation of Aβ LTMRs. **e**, Number of evoked spikes per pulse in each driver line for VPL units. Markers represent individual units and bars indicate medians. Yellow-shaded region represents range of values observed in Aβ LTMRs. Two-sided Mann-Whitney U=894.0.

## Data Availability

Data are available upon request to the corresponding authors. Source data are included.
